# Tripping over my EGO: how a starving cell taught me to survive

**DOI:** 10.1093/femsyr/foag018

**Published:** 2026-05-15

**Authors:** Claudio De Virgilio

**Affiliations:** Department of Biology, University of Fribourg, Chemin du Musée 10, CH-1700 Fribourg, Switzerland

**Keywords:** trehalose metabolism, TORC1, Rag GTPase, EGO complex, growth control, nutrient sensing, cellular quiescence, Rim15, vacuole Saccharomyces cerevisiae

## Abstract

This retrospective traces my scientific journey from my childhood in the working-class St. Johann district of Basel, to my unexpected contributions to the field of cellular signaling and nutrient sensing. Framed by early struggles with identity as the son of an Italian immigrant and a turbulent search for academic belonging, my path to scientific independence was anything but linear. I candidly recount my detours—repairing shoes, filling car batteries with acid, and hauling ladders as a field biologist—before finally finding my true calling in the quiet precision of the laboratory. The narrative highlights the often-serendipitous milestones in my career, beginning with early graduate work that challenged prevailing assumptions to establish the simple sugar trehalose as a critical cellular survival factor. It follows my transformative, though initially humbling, postdoctoral years in the United States, a period of acute professional uncertainty upon returning to Switzerland, and my gradual rise to independence. Through every triumph and setback, this story is anchored by my love and partnership with my wife, Michèle—my grounding counterpoint and the steadfast constant in a life of shifting scientific models. Scientifically, the narrative culminates in our laboratory’s collaborative successes: identifying Rim15 as the integrator of PKA and TORC1 signalling that controls the entry into cellular quiescence and uncovering the EGO/Rag GTPase complex—the vacuolar command center that activates TORC1 to drive the exit from this dormant state—as a universal blueprint for eukaryotic nutrient sensing. By reflecting on how our fundamental genetic discoveries in yeast translated into clinical interventions for human diseases, this essay illustrates the unpredictable yet immense value of basic science. More than a chronicle of academic achievement, this is a human story exploring the intense pressures of the scientific enterprise, the importance of mentorship and personal connection, and the enduring power of curiosity-driven research.

## Teaser

Retrospective on the work of Claudio De Virgilio.

## The industrial triangle—the gate, the hidden science, and the prison

### TOR, the gate

**Figure 1:**
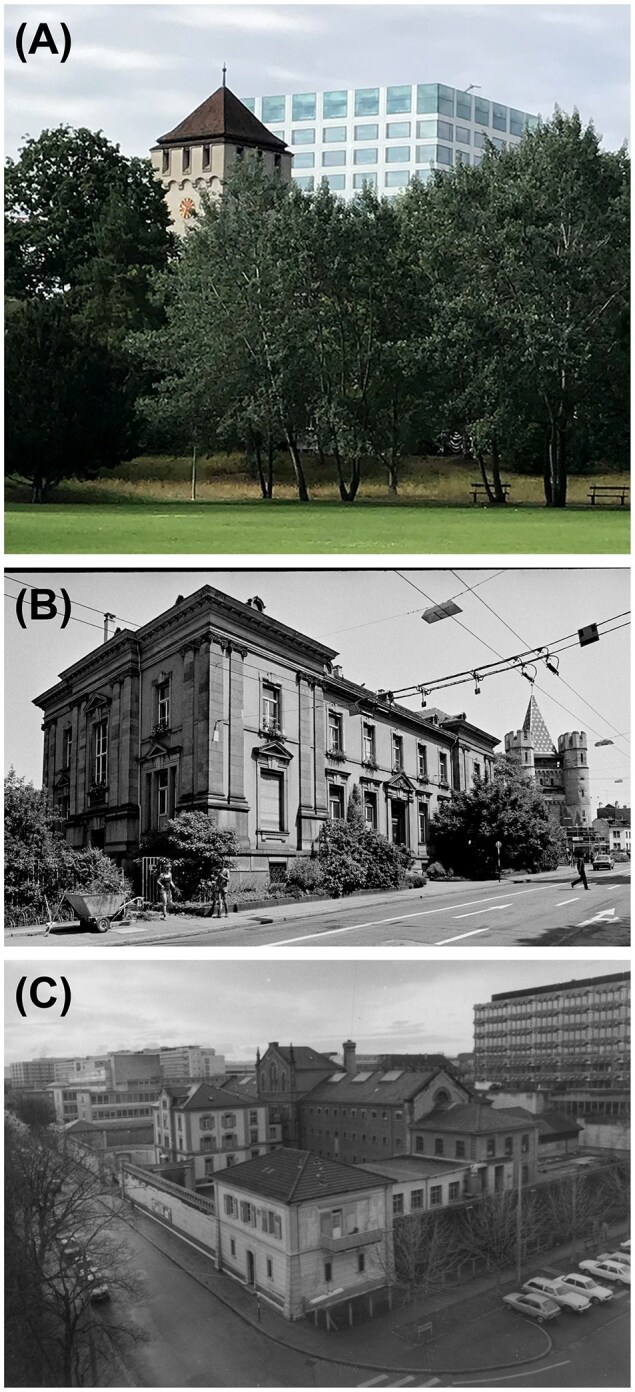
(A) The St. Johanns-Tor and the new Biozentrum: a modern view of the “Industrial Triangle” where the medieval gate and the future of TOR signaling converge on the former grounds of the Schällemätteli prison. (B) The Botanical Institute stands prominently in the foreground, with the Spalentor—the medieval gate that partly inspired the name of the TOR protein—in the background. It is a poetic coincidence that my future lay not with the famous gate, but in the building right in front of it. Photo: Claude Giger/SozArch F 5156-1981-0736-31. (C) The grim walls of the Schällemätteli prison, with the Biozentrum rising in the background. This photograph was taken directly from the building of my primary school, the St. Johanns-Schulhaus. Just out of frame, immediately adjacent to the trees on the left, sat our soccer field. Photo: *Urheber:in unbekannt*/SozArch F 5185-Fb-320.

Retrospectives often begin in sterile laboratories or hushed lecture halls, but my scientific journey began on the streets of a working-class district in Basel, long before I knew what a pipette was. I was born in 1964, a time when the St. Johann district was defined not by the gleaming glass of modern biotech campuses, but by the heavy, sweet-chemical scent of industry and the soot of the *Arbeiterquartier* (workers’ quarter). To understand my origins, you must visualize a specific triangle in the urban geography of 1960s Basel. My world was bordered by three landmarks that, in hindsight, seem almost too symbolic for my life as a future scientist. To one side stood the St. Johanns-Tor, a medieval gate standing silent watch over a modernizing city (Fig. 1A). By a strange quirk of history, the protein kinase TOR (Target of Rapamycin), which has defined so much of our field, owes its name to a gate in Basel. Joe Heitman (working in Michael Hall‘s lab at the Biozentrum) and Sandoz scientist Rao Movva, who collaborated to discover TOR, named the protein partly to honour the Spalentor (Fig. 1B) (Heitman 2015). However, from the perspective of a local boy, the geography of discovery points elsewhere. The Biozentrum is only 200 m away from the St. Johanns-Tor. Had the wind blown differently, we might be studying a target named after this gate of the working class, rather than the more famous gate.

### The secret in the cellar

To the other side lay the Institute of Physics, housing a spectacular secret: the AGN-211-P nuclear reactor. A former star of the 1958 World Expo, this icon of the atomic age was moved from the world stage to a humble cellar in St. Johann, operating just 50 m from where I slept. Science was already buzzing beneath the floorboards of my childhood, even if I couldn’t see it yet.

### From prison to laboratory

**Figure 2:**
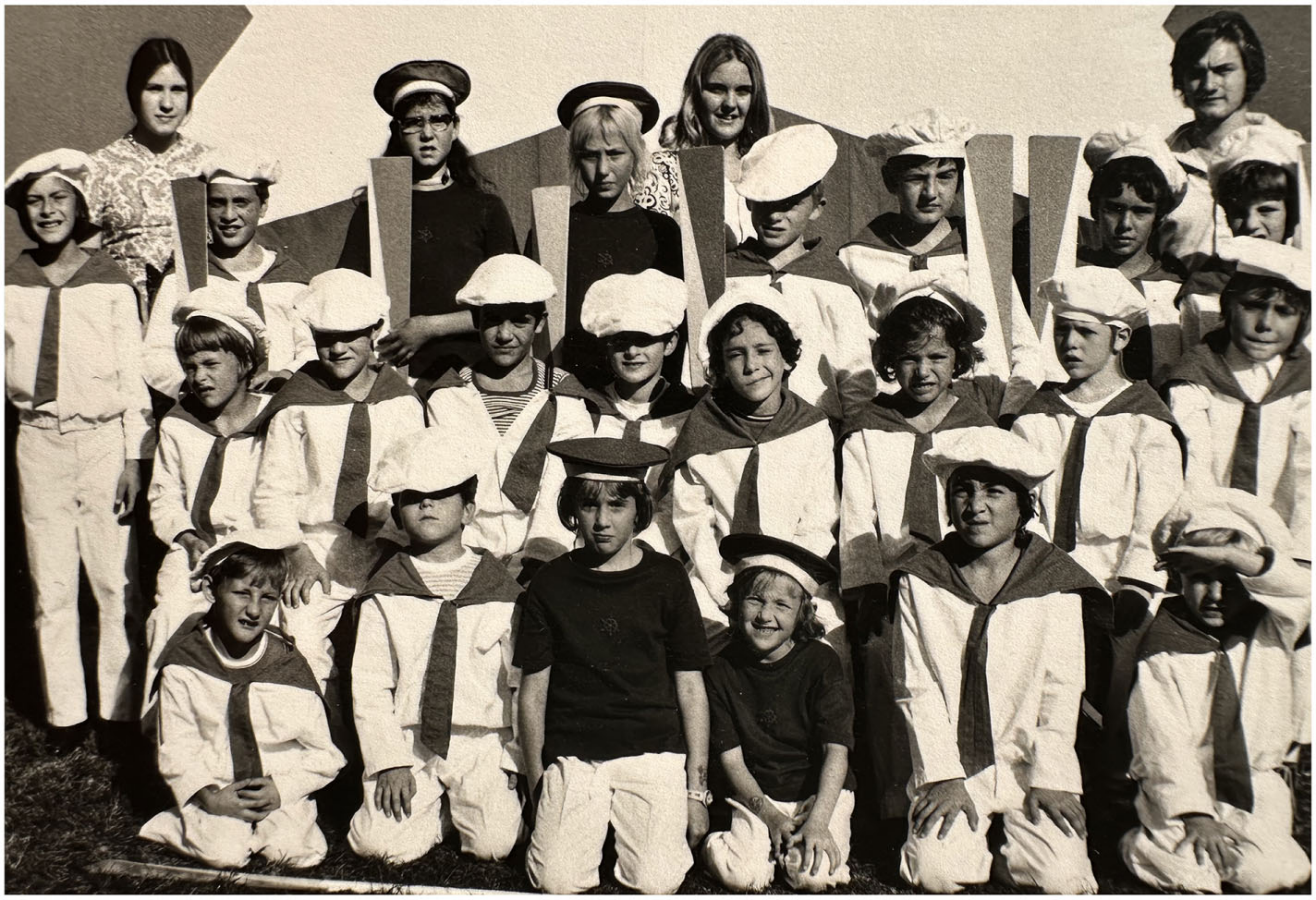
A true “local boy” (front row, second from the right) in a sailor suit at the 1974 St. Johanns-Fest, standing on the St. Johanns-Schulhaus soccer field with the district’s historic cross of St. John in the background.

Completing this triangle was my daily center of gravity: the St. Johanns-Schulhaus and its football field (Fig. 2). We played there until sunset, barely acknowledging the grim walls of the Schällemätteli prison looming just across the street. We had no idea that the future was being constructed behind those walls; the Biozentrum was rising on those very penitentiary grounds (Fig. 1C). I was literally playing in the shadow of the place where the molecular basis of the TOR pathway would eventually be unravelled.

### The son of the *Gastarbeiter—*good enough for a red passport?

**Figure 3:**
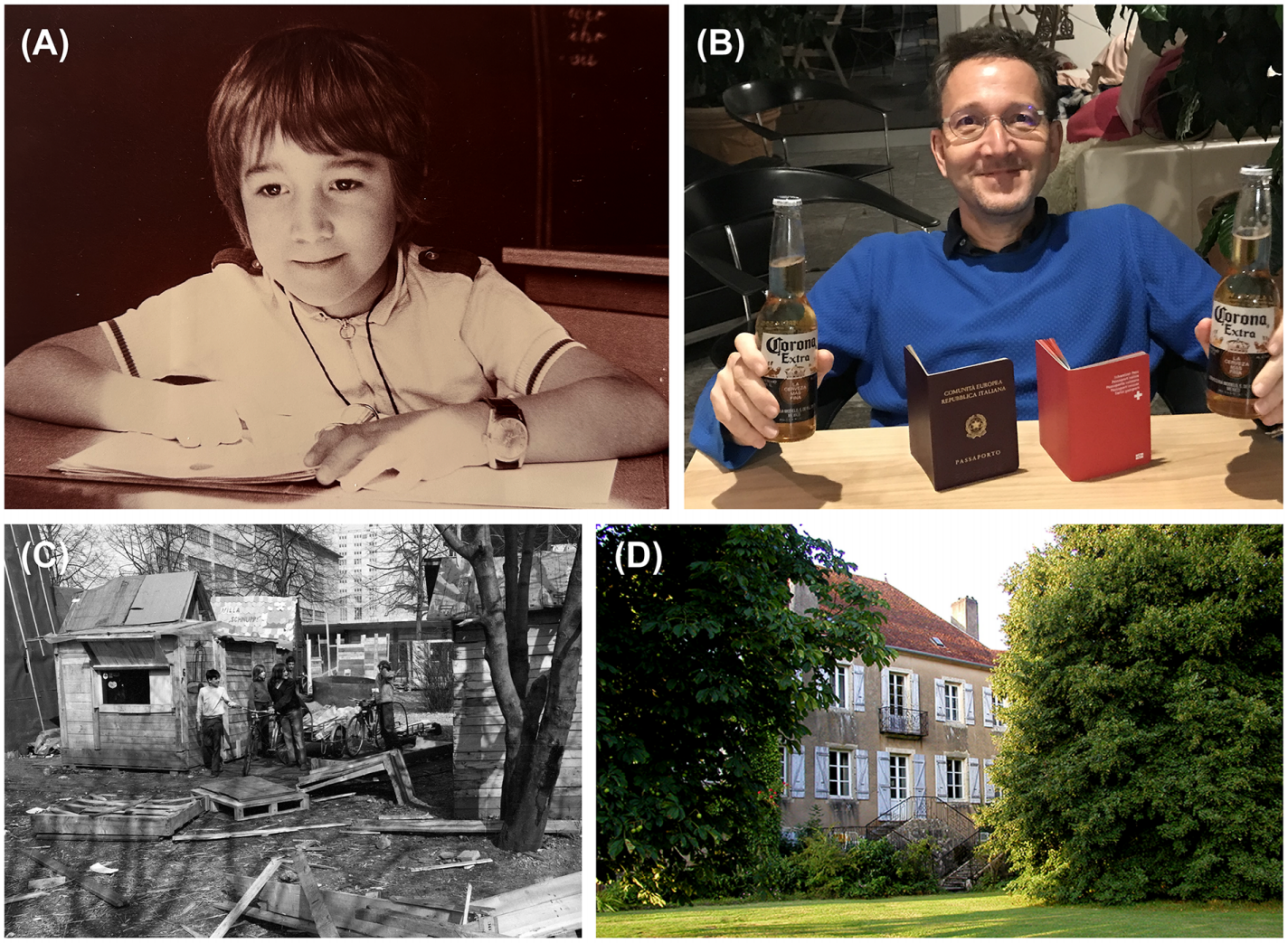
(A) Dreaming beyond the page at primary school. (B) The genetics of my identity: definitively heterozygous for citizenship. Celebrating my dual roots and maintaining the equilibrium with a Corona in each hand. (C) The Robinson-Spielplatz Volta, where we built our makeshift huts. The multi-story industrial buildings looming directly in the background belong to the former Sandoz complex, a site that, following the merger with Ciba-Geigy, would be transformed into the modern Novartis Campus. In a twist of fate, the building visible on the far left is exactly where I now attend meetings as a board member of the Novartis Foundation for Medical and Biological Research, a position I have held since 2015. Photo: Heinz Bruni/SozArch F 5146-Fa-0889. (D) The chateau in the Franche-Comté: a retreat where science and art converged.

In the 1960s, that football field was less about the future and more about survival in the present. St. Johann was a neighbourhood of contrasts, the era of the *Gastarbeiter—*the “guest workers” recruited from Southern Europe to fuel the country’s economic boom. My father Cosimo was an immigrant who had first moved from Puglia to Bologna to find work as a tailor before settling in Basel; my mother Charlotte (“Lotti”) was Swiss, a factory worker who assembled electric engines. Because both of my parents worked demanding hours, I became a *Schlüsselkind* (latchkey child) long before the age of six (Fig. 3A). Growing up as the son of an Italian immigrant meant reconciling a complex identity. I was often branded with slurs like “Tschingg,” (Italian immigrant). It made me feel like an outsider in my own city (a striking historical contradiction, considering the St. Johann district itself traces its name back to the Order of St. John, founded by Italian merchants from Amalfi, which established a presence in Basel in 1206, long before the city even joined the Swiss Confederation in 1501). This tension culminated in a process that felt lifted directly from the satirical film *Die Schweizermacher*. I vividly remember federal agents inspecting my performance at primary school or sitting in our kitchen checking if we were “Swiss enough” to deserve the red passport. We were finally naturalized, but due to a clerical error at the Italian Embassy—my renunciation papers were lost—I unknowingly also remained Italian. I only discovered this years later, by which time Switzerland had legalized dual citizenship (Fig. 3B). In essence, my childhood as a *Schlüsselkind* and those early experiences of scrutiny instilled in me a fierce independence, a trait that serves a scientist well when data contradicts dogma.

## The Robinson remedy and industrial shadow

### From arena to battlefield to refuge

While the football field was my arena, home was often a battlefield. My parents’ marriage dissolved in a volatile, loud crash that saw my father’s clothes thrown onto the street. The ensuing divorce brought no peace; my mother remarried a man who struggled with alcoholism. Home became a place to escape from. Seeking respite from a turbulent home, my brother and I found refuge in the Robinson-Spielplatz Volta. Bordering the industrial walls of Basel, it was an island of unbridled liberty. Unlike modern, sanitized playgrounds, this was a place of scrap wood, hammers, and dirt (Fig. 3C). It was here, amidst the “wild west” atmosphere and the looming pharmaceutical factories, that I learned to build things with my hands and, vital for a scientist, how to calibrate risk. The reality of this environment was often stark; I once stepped on a rusty nail and developed sepsis, surviving only thanks to antibiotics likely produced in the very factories casting shadows over our makeshift hut. At the time, Sandoz and Ciba-Geigy (later merged to become Novartis) were simply where the neighbors worked. I could not have known then that inside those very Sandoz buildings, researchers would one day provide the rapamycin expertise that, just down the street at the Biozentrum, would unlock the science of my future. Looking back, it seems almost too poetically fitting that my survival as a child depended on the industry whose most famous molecule would eventually become the centerpiece of my life’s work.

### From safe haven to rebellion to the prize

When I moved in with my father and my future stepmother, Ursula, after primary school, the domestic volatility that had marked my early years finally settled. For the first time, I had the mental space to dedicate myself to my studies. I didn’t just tolerate school; I used it as a shield. In the textbooks, everything was logical, clean, and explainable, a stark contrast to the messy unpredictability of my parents' divorce. This early immersion in structured thought provided the basis for my future life in the laboratory. However, this academic fortress was not immune to the political currents swirling through Basel in the late 1970s. While the city was in a “gestation” phase of a youth movement, with many of my peers taking over specific sites like the old post office and the *Kaserne* (the former military barracks) to claim autonomous spaces, the pressure to conform to this new rebellion was heavy. This anarchy went entirely against my nature. Perhaps because of the resilience I had developed to weather childhood chaos, I found myself craving structure rather than revolution. I refused to participate in the AJZ (Autonomous Youth Center) movement, resisting the peer pressure to rebel against the mainstream. In a sense, I found my own form of rebellion in stability (De Virgilio 2026a, https://doi.org/10.5281/zenodo.19372184). I finished high school with a spirit of determined autonomy and a graduation prize in my pocket. I took that money and invested it in a symbol of the intellectual order I craved: the *Lehrbuch der anorganischen Chemie* by de Gruyter. Armed with this seminal text, I was ready for university.

## The Vatican, the prison, and the speed of light

### From a dream to a nightmare

In 1983, the Biozentrum of the University of Basel was in what many consider its “Golden Era”. It was the Vatican of molecular biology, housing Nobel Laureate Werner Arber and legends like Walter Gehring and Jeff Schatz. Seeking the frontier of the field, I enrolled in “Biology II”, the modern, elite track, convinced this was where I belonged. The disappointment, sadly, was immediate. Instead of the biological wonders I expected, I was met with a wall of mathematics, physics, and physical chemistry. Even more troubling than the curriculum was the atmosphere; for me, it was not one of collegial discovery, but of cutthroat competition and intellectual detachment. It felt surprisingly restrictive, more like a penal institution where signatures were collected to prove our presence.

### It was observation not prediction

The turning point arrived in a physics practical. A colleague and I measured the speed of light with such precision that the assistant professor refused to believe the data. Convinced we had “predicted” the result rather than observed it, he denied us the credits. To a young student, this was fundamentally disturbing. I had sought science for its objective truth, but found myself manoeuvring through a system where authority mattered more than the evidence before one’s eyes. Recognizing that my scientific journey required a different kind of discipline, I walked away after just one semester.

### Leather and acid

**Figure 4:**
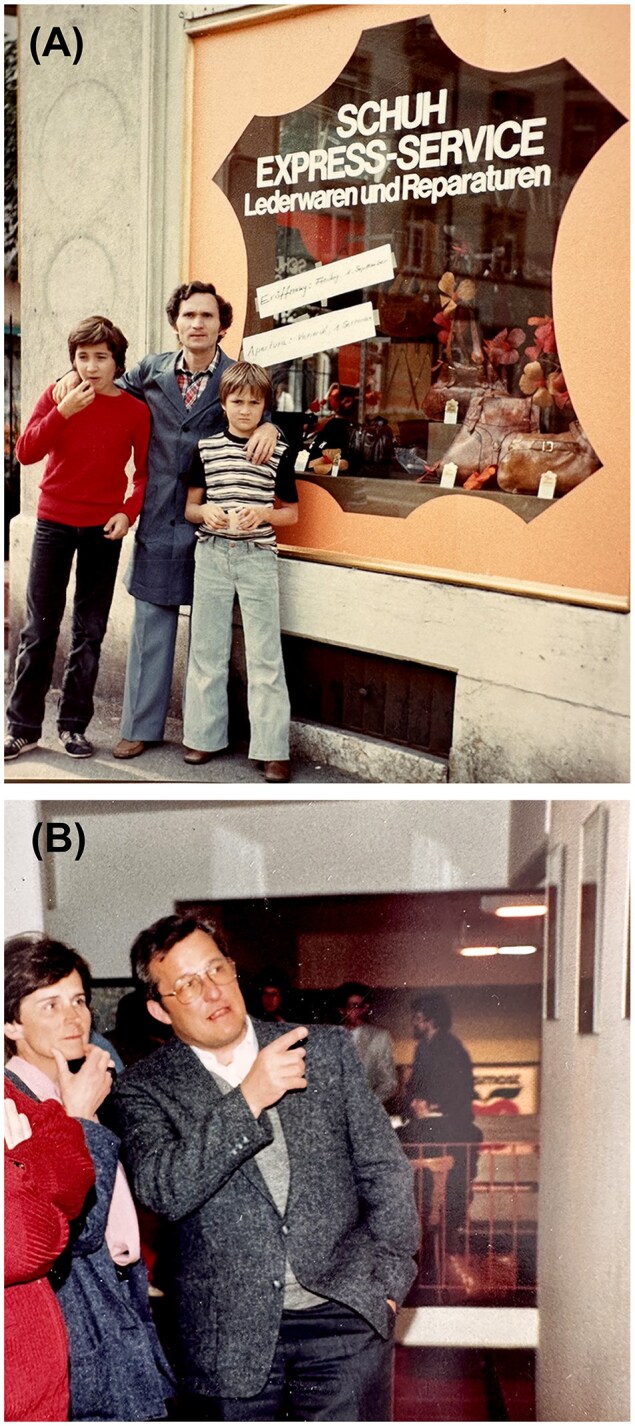
(A) 1 September 1978: standing with my younger brother Silvio and my father on the opening day of his shoe repair shop in St. Johann, the *Arbeiterquartier*. (B) Heinz Durrer and his wife discussing my art at my tennis center vernissage (with me, the blurry figure in the background over the shoulder of Heinz). A brief dream of becoming a tennis champion was officially abandoned at that time.

Following my departure from the university, I retreated to the world I knew: the working class. My father had become a cobbler in St. Johann, and for three months, I worked in his store repairing shoes (Fig. 4A). He was a role model of a relentless work ethic who openly showed his love and supported whatever path I chose. His unconditional pride gave me much-needed inner stability during an uncertain time. I was good with my hands, a skill honed years earlier amidst the scrap wood of the Robinson playground, but there wasn’t enough work for two men, and I felt unfulfilled. I moved on to Leclanché, a battery company. My mornings were spent filling car batteries with sulfuric acid, a dangerous, pungent task, before delivering them to customers in the afternoon. It was honest work, but as the acid fumes stung my nose, I realized this could not be the end of my story.

### Diverging from the abstract—my final brush with art

During the summer of 1984, while completing my mandatory military service, I made one last attempt to diverge from science. I had always painted to escape, even managing a vernissage at a tennis center where I sold a few pieces (Fig. 4B). I applied to the *École Supérieure d’Art Visuel* in Geneva. I prepared my portfolio on the rare Sundays off from the army, exhausted but hopeful. While my portfolio was well-received—the committee encouraged me to resubmit the following year because all seats for the current intake were already filled—attending simply became unfeasible. I couldn’t afford to wait, nor could my parents afford to support me financially in Geneva. As the door to art closed, it left me with one clear option: I decided to pick up my studies again. But this time, I turned my back on the Biozentrum and opted for “Biology I”, classical biology. This meant leaving the sterile labs for field courses, ecology, evolution, and the systematics of plants and animals. I didn’t know it yet, but I was trading the abstract for the concrete, a move that would finally ground my scientific journey and provide the organismic perspective of my research.

### Life, death, and epiphany on a vertical marathon

My move to “Biology I” finally delivered the tangible experience I had craved, though it often manifested in surreal ways. I joined a conservation project led by Heinz Durrer and Urs Tester, aiming to resurrect the region’s extinct tree frog (*Hyla arborea*) population. At the time, scientific consensus held that once a local amphibian population was extinct, it was gone forever. Durrer and Tester set out to prove otherwise, creating a network of “stepping-stone” ponds to encourage natural recolonization. Oddly enough, my role in this rebirth played out in the cellar of the Institute of Pathology, directly next to the cold storage for cadavers. There, amidst the scent of formalin and mortality, I bred the flies needed to feed our tadpoles, once even chasing a cloud of escaped insects through the sombre halls. Nights were spent in the wetlands of the *Petite Camargue Alsacienne* in France, waiting in the darkness to triangulate the metallic calls of the frogs. We proved the population could return, but the work was a lesson in the stark realities of nature. If the frogs taught me patience, the Great Tits (*Parus major*) taught me my limits. I joined a project funded by the Swiss National Science Foundation (SNF) under Arie van Noordwijk with the imposing title: “*The effects of environmental heterogeneity on the expression and ascertainment of genetic (co-)variation”*. The scientific logic was elegant: we were investigating how the chaotic reality of nature, the “environmental heterogeneity”, masks or reveals the true genetic potential of a population. To disentangle nature from nurture, we needed precise data from the steep terrain of a mountain ridge called Blauen. The reality, however, was a brutal vertical marathon. I hauled a cold aluminium ladder up a 400-m ascent daily, dodging suspicious dogs and checking nest boxes that sometimes housed angry hornets instead of birds. The breaking point arrived on my very last day, when a thunderstorm broke over the summit, soaking me to the bone. Standing there, shivering and muddy, clutching my ladder, I had an epiphany: I respected the data, but the arduous life of a field biologist was for others. I was ready to return to the lab.

### Nobody cares about you

My decision to step out of the rain was also heavily shaped by the intellectual environment indoors, particularly the influence of Stephen Stearns, the head of the Institute of Zoology. Long before he became a renowned professor at Yale University, Stearns was already a towering figure in our field, actively laying the cornerstones of life-history evolution and co-founding the European Society for Evolutionary Biology right there in Basel. In 1987, he penned his now-legendary manifesto, “Some modest advice for graduate students” (Bulletin of the Ecological Society of America, 1987; Volume 68, pages 145–150). I took his brutally honest maxims to heart, particularly his stark insistence that “Nobody cares about you”. His point was to emphasize that you, and only you, must take absolute ownership of your scientific destiny. Leaving the mountain ridge wasn’t just about fleeing the mud; it was my first act of doing exactly that.

### Are you not selective?

The Zoology building itself, located right on the Rhine, fostered a brilliantly provocative atmosphere. I vividly remember a small-group seminar held in a wonderfully atmospheric room where the pioneering Renaissance anatomist Andreas Vesalius was said to have prepared his historic skeleton during his time at the University of Basel in 1543. During one session, we were debating the classic evolutionary theories of sexual selection. I confidently argued the prevailing established paradigm of parental investment: because females invest vastly more energy into producing eggs compared to the “cheap” sperm of males, females are inherently the choosy, selective sex, while males indiscriminately compete for access. Stearns let me finish my grand, textbook defense before leaning in and asking in front of the class, “Are you not selective, Claudio?” In an instant, the abstract theory collapsed into personal reality. It was a humbling lesson in questioning underlying assumptions and realizing that elegant biological models often mask complex realities, exactly the mindset I needed as I finally traded the field for the bench.

### An invitation to the bench—I found my element

Rain on the Blauen had washed away my romantic notions of field biology, but it cleared the path for a different kind of discovery. I traded the mud for the bench, finding my true calling indoors under the mentorship of Helene Kuhn, a postdoctoral fellow at the Institute of Botany. She allowed me to contribute to her research on the biochemical interplay between soybean plants (*Glycine max*) and the pathogenic fungus *Phytophthora megasperma*. We focused on ornithine decarboxylase (ODC), a rate-limiting enzyme pivotal for polyamine biosynthesis. Our experiments were designed to dissect how this enzyme was regulated during the plant’s immune response, a cascade that finally triggers the production of glyceollin, the potent phytoalexin the soybean uses to poison the invader. I was captivated. Unlike the unpredictable chaos of the field, here we could manipulate physiological variables and measure the enzymatic response with kinetic precision. Watching those biochemical pathways reveal themselves, I realized that I didn’t want to just observe nature’s patterns; I wanted to understand the machinery driving them. In the steady, controlled hum of the laboratory, I knew I had finally found my element.

### Time stopped—I found my home

1988 brought another discovery, one that would prove more defining than any data point. It was a warm summer day, and I was sitting on the banks of the Rhine, where students gathered for lunch. A colleague introduced me to Michèle. As the break ended, everyone packed up to return to class. I was scheduled for a plant physiology practical, but looking at Michèle, the complex mechanisms of photosynthesis suddenly seemed utterly irrelevant. In a rare departure from my usual discipline, I skipped the practical. We stayed by the river all afternoon, lost in a conversation that didn’t just pass the time, it stopped it. From that day on, we were a team. Michèle became the invaluable counterpoint to my scientific life, often joining me during long nights and weekends in the lab. She would sit at a nearby bench, immersed in Shakespeare while I ran time-points, occasionally peering through the microscope to share in the wonder of the invisible world I was exploring. In those quiet hours, amidst the drone of machinery and the pages of her books, I realized that while science was my passion, she was my destination. In that laboratory, I had found my home.

### The chateau, papers, and surrogate family

While I had found my anchor in Michèle, she had been forced to learn self-reliance long before we met. Her childhood was marked by an uphill battle of its own: her mother had left the family when Michèle was very young, and she was raised by her father. When he passed away, leaving her effectively on her own at the age of 17, she found a safe haven in Steffy and Peter Dukor. Steffy was a close friend of her father’s, and she and Peter became a surrogate family to Michèle and later to both of us. Peter was a formidable figure in science, a renowned immunologist who had done pioneering work on the thymus. He had spent years at Ciba-Geigy in Basel before becoming the Director of the Sandoz Research Institute in Vienna. They owned a small chateau in the Franche-Comté (Fig. 3D), and for Michèle and me, this place became a sanctuary. We spent many weekends and summers there, immersed in a world that felt suspended in time. It was a place of enriching contrasts: Steffy would invite artists (musicians and painters), while Peter brought in scientists from his company. These gatherings were my first true window into the human side of the industry. I listened to researchers debate not just data, but life, dissolving the rigid caricature of the “industrial scientist” I had held since childhood. While the guests debated, I often retreated to a tranquil corner of the chateau with a stack of papers I had photocopied from the Biozentrum library. In those pre-digital days, the literature was still small enough that one could master a field by reading 20 or 30 key papers in a summer. There, surrounded by the French countryside and the faint murmur of intellectual conversation, I honed the art of critical reading. When Peter died in 1995, it was an monumental loss for us both. But those summers in the Franche-Comté gave us something permanent: a model of how science, art, and friendship can flourish under one roof.

### The intricate dance

In 1989, I began my PhD studies in the laboratory of Andres Wiemken and Thomas Boller (Fig. 6A) at the Institute of Botany in Basel. It was a place where the boundaries between plant and fungal physiology were productively blurred, and where the scientific lineage ran deep. The two heads of the lab shared a long history of collaboration, often uniting their expertise to study the intricate dance between plants and fungi, from the symbiotic networks of mycorrhiza to the chemical warfare of innate immunity. This interconnectedness extended to the very architecture of the cell.

### Paradox resolved

Years prior, Wiemken had collaborated with future Nobel Laureate Paul Nurse to isolate the contents of the vacuoles from *Candida utilis*, a pioneering effort in cellular compartmentalization. Wiemken had uncovered a fascinating cellular logic: acidic trehalase was sequestered in the vacuole, while its substrate, trehalose, resided in the cytosol. This spatial separation prevented a futile cycle, keeping the fuel safe from its own breakdown machinery during starvation. However, yeast cells also massively accumulate trehalose in the cytosol during heat stress, which posed a distinct functional puzzle. The regulation of the cytosolic neutral trehalase, which shares the same compartment as the accumulating sugar, remained unresolved. Although the enzyme was known to be activated by phosphorylation, its physiological state during heat stress was obscured by extraction artifacts. In one of my first contributions to science, I resolved this paradox by developing a rapid permeabilization assay. We proved that the cytosolic enzyme avoids a futile cycle through strict temporal regulation: it remains safely dormant while the cell accumulates trehalose during the heat shock and is rapidly activated by phosphorylation only upon recovery when the sugar is no longer needed.

### The balance of vision and verification

**Figure 5:**
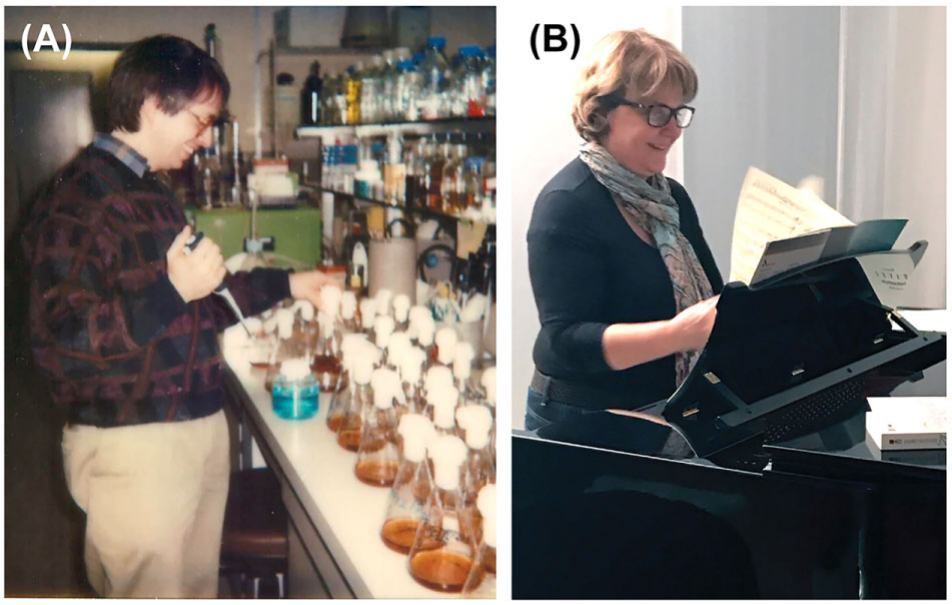
(A) Thomas Hottiger (left) conducting his research across an intimidating fleet of Erlenmeyers, while Beate Westenberg (B) 25 years later, gracefully orchestrates symphonies at the piano.

Deciphering such physiological complexities required a balance of creative questioning and methodological rigor, precisely the duality that defined my daily life in the lab. On one side was Thomas Hottiger (Fig. 5A), a scientist of boundless energy and intellectual dynamism. It was Thomas who challenged the prevailing view that trehalose was merely a storage carbohydrate for lean times. Through sheer innovative force, he demonstrated that yeast cells accumulated this sugar as a shield against heat shock, redefining it as a critical stress protectant. On the other side was Beate Westenberg (Fig. 5B), who embodied methodical precision and structural clarity. While Thomas pushed boundaries, Beate provided the exacting framework, unravelling the metabolism of polyphosphates in those same vacuoles with systematic exactitude. They created the ideal ecosystem for my growth. I learned to balance Hottiger’s creative vision with Westenberg’s disciplined verification, synthesizing a scientific identity that drew power from both. Looking at where their paths led, Thomas bringing his energetic brilliance to the highly structured world of quality control at Swissmedic (the Swiss equivalent of the FDA), and Beate channelling her meticulous dedication into a career as a professional pianist, is a wonderful reminder that the best scientific minds are wonderfully multifaceted. Beyond the bench, my connection with Beate blossomed into a lifelong family bond; through a remarkable series of serendipitous overlaps, we remain close friends to this day, with Michèle proudly serving as the godmother to Beate’s daughter. Under the tutelage of Thomas and Beate, I began to see that the humble yeast cell was not just a model for baking bread, but a masterclass in survival strategy.

## Heat, dogma, and a FAX from Olympus

### Yeast cells that forgot to read the literature

My own contribution to this masterclass in survival began in the weight of a formidable, and at the time, immovable dogma. These were the defining years of my PhD, where I quickly learned that in the high-stakes world of molecular biology, the weight of a name can sometimes briefly obscure the weight of the data. At the time, the consensus held that heat shock protein 104 (Hsp104) was the sole master of thermotolerance. However, the yeast cells in our hands apparently hadn’t read the literature; working with strains from the laboratory of Susan Lindquist, we observed that they survived severe heat shock quite happily without Hsp104, provided they were well-stocked with trehalose (De Virgilio et al. 1991).

### Of course, we knew it all of the time

We published our findings, proposing that this simple sugar acted as a critical survival factor. We explicitly outlined a dual model for acquired thermotolerance: we suggested it relies on two independent mechanisms, with trehalose providing “induced protection” during severe heat stress to prevent damage, while heat shock proteins handle the “induced repair” of damage that has already occurred. The response was unusually sharp. On 17 October 1991, a fax arrived from Susan Lindquist herself, the reigning titan of the field, informing me that we had published “erroneous conclusions” and had essentially missed the mark. As a PhD student, receiving such a “thunderbolt from Zeus” was a terrifying rite of passage, yet I found myself in the curious position of having to choose between the leader of the field and my own results. I chose the results. Scientific redemption arrived a mere twenty-four hours later, virtually at the speed of light usually reserved for the physics department, via a letter from Michael P. Yaffe. His lab had independently found a mutant that acquired thermotolerance despite a defect in heat shock factor 1 (Hsf1), the transcription factor required for Hsp104 expression, and he explicitly preferred our “chemical chaperone” explanation over the prevailing theories. In a familiar twist within our trade, years later, the Lindquist lab published this exact concept, outlining the precise dichotomy of trehalose acting first to stabilize proteins against damage, and heat shock proteins acting later to promote refolding. Rather than framing this as a validation of our 1991 model, they cited the prior literature as a “long-standing subject of controversy” that their new data had finally clarified. I take it as a compliment; after all, in science, being called “controversial” is often just the first stage of being proven right.

### The cold room and the midnight peak

To prove our model definitively, we needed genetic control. We needed to clone the genes responsible for making trehalose. But before we could get the gene, we had to wrestle with the protein. The path had been cleared by Walter Bell, a colleague who had established the gruelling purification protocol and successfully identified the gene encoding the 56-kDa subunit Tps1, which acts as the trehalose-6-phosphate synthase. Building on Walter’s initial progress, I spent endless days and nights shivering in the cold room, watching fractions drip through column after column to isolate the rest of the complex. It was a test of physical endurance, but it paid off. We had the purified material microsequenced at the Biozentrum using automatic Edman degradation. That sequence was our map, leading us directly to the 100-kDa subunit and the *TPS2* gene (De Virgilio et al. 1993).

### Where exactly is my plasmid?

However, identifying the gene was only half the battle; to prove its function, we had to delete it. At this point, I hit a technical wall. While physiology was my home turf, the molecular biology required for a gene deletion was, at the time, a foreign language to a student at the Institute of Botany. I was essentially “learning by doing” from textbooks, often with messy results. My saving grace was Patrick Linder at the Biozentrum, who, having already discovered the ubiquitous DEAD-box family of RNA helicases, carried with him the stringent genetic legacy of his postdoctoral mentor, the legendary Piotr Slonimski. In a wonderful display of the collegiality that defines the best of our community, he took a “plants” student under his wing. I still look back with a smile at our first meeting, where I presented him with a Polaroid of a blurry agarose gel and asked, in all seriousness, “Patrick, where exactly is my plasmid?”. Instead of the laughter I probably deserved, he offered patient guidance that not only allowed me to construct the *tps2* mutant but also set the course for my future career.

### The feeling I have been chasing ever since

The true “Eureka” moment followed in the solitude of the lab on a peaceful night. I injected the cell extract from the mutant into the HPLC and watched the pen on the chart recorder. The familiar trehalose peak never came; instead, a new, unknown peak emerged earlier in the run. In an instant, the chemistry clicked: I had deleted the phosphatase, and the cell was now piling up the intermediate, trehalose-6-phosphate. Sitting there in the steady rhythm of the instruments, I had a visceral realization: biology was not just descriptive, it was programmable. That addictive high of unlocking a secret mechanism is a feeling I have been chasing ever since.

## The Vienna connection and the next leap

### Behind every data point lies a human being

**Figure 6:**
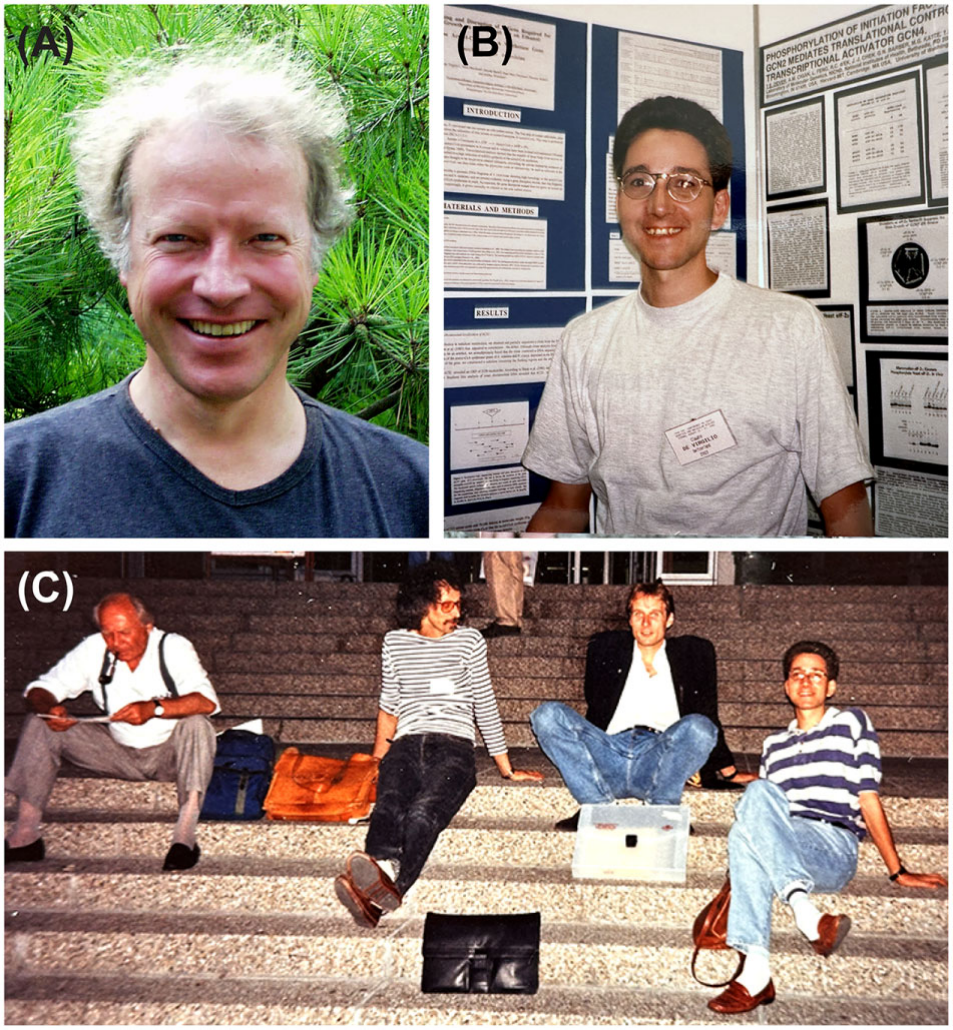
(A) Thomas Boller, photographed in 2009 in front of the *Hebelstrasse* greenhouse. Alongside Andres Wiemken, he was a guiding mentor during my PhD and supporter during my transition to scientific independence. (B) Proudly presenting my *ACS1* cloning work, completely unaware of the tearful encounter with a fellow student that was about to unfold in the afternoon. (C) Vienna, August 1992: on the stairs after the ICYGMB16 poster session (from right: me, Niels Bürckert, Patrick Linder, and the legendary Piotr Slonimski), honoring the scientific legacy Patrick first forged as a postdoc in Slonimski’s lab.

In August 1992, the next chapter of our lives opened in a poster hall in Vienna during the 16th International Conference on Yeast Genetics and Molecular Biology (ICYGMB) (Fig. 6C). It was a pivotal moment, providing a poignant lesson in the human dimensions of our trade. My primary contribution was a poster describing the cloning and analysis of the *ACS1* gene coding for the acetyl-CoA synthetase (Fig. 6B). While I viewed it as a solid piece of work, I arrived at the session to find a PhD student in tears before my board; the cloning of *ACS1* had been her entire thesis project. I spent a long time speaking with her, emphasizing the unpredictability of discovery rather than the spirit of competition, but the encounter left a deep mark. It was a stark reminder that behind every data point lies a human being. That encounter tempered my ambition with empathy. It taught me that while science is often a race, the people running it matter more than the finish line. I walked away with a conviction: success should never be measured solely by who gets there first, but by how we treat our colleagues along the way.

### YES! welcome to the major leagues

**Figure 7:**
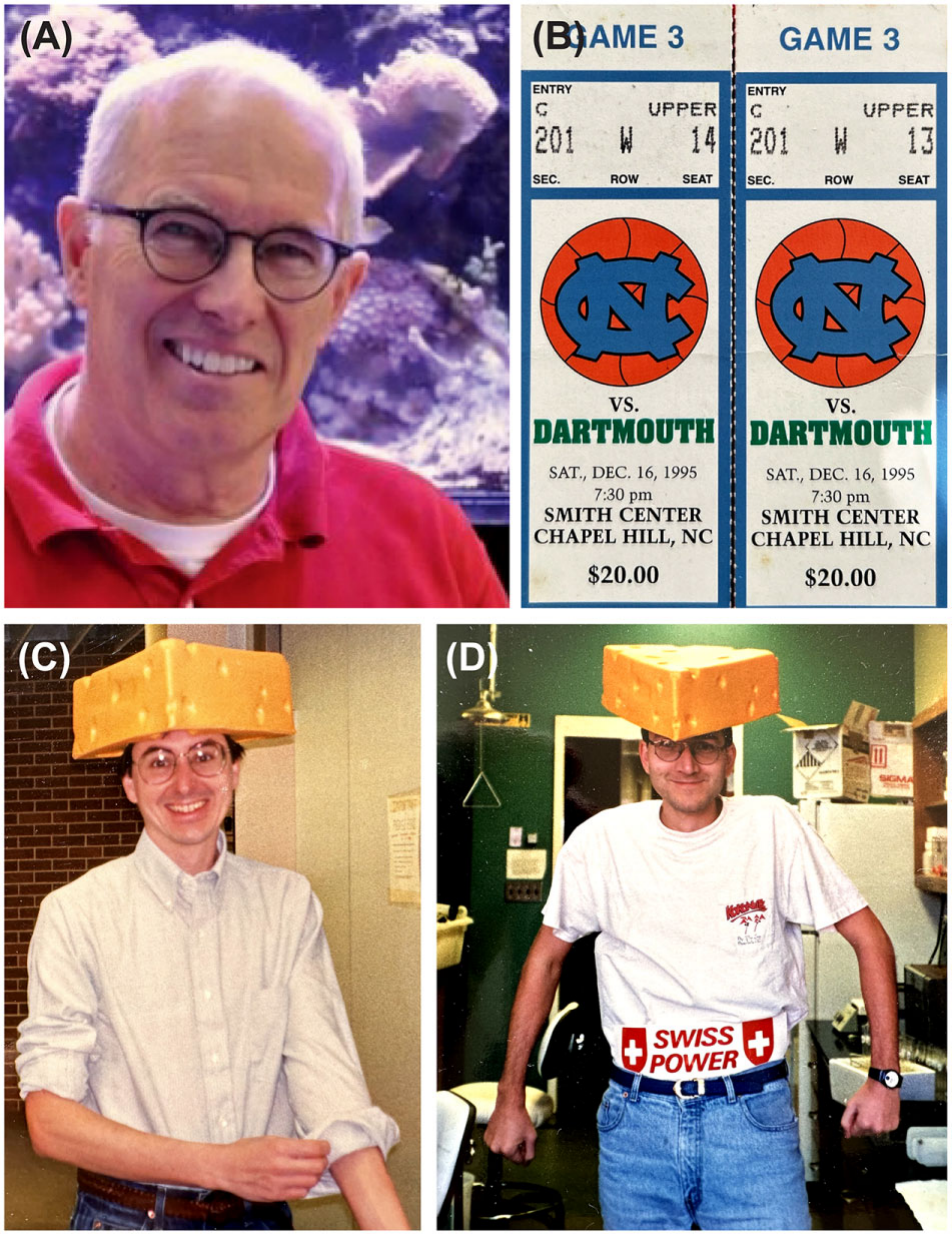
(A) John Pringle, the mentor who drafted me into the major leagues and taught me the Olympic standard of science. (B) The spoils of our greatest non-scientific victory: the tickets from our legendary Dean Dome “heist” to watch the Tar Heels play. (C) My benchmate Doug DeMarini in his natural Wisconsin “cheesehead” habitat at Fordham Hall in Chapel Hill. (D) Me returning the favor with a “Swiss Power” teaser: a snapshot of the “insider jokes” and joyful camaraderie that made our time in the Chapel Hill “Hot-lab” the best years of our lives.

Turning from this encounter, I went to stand by my second poster detailing our trehalose findings. There, I saw a figure approaching who needed no introduction: John R. Pringle (Fig. 7A), the UNC luminary of the yeast cell cycle field who had trained as a postdoc under future Nobel Laureate Leland Hartwell. To my surprise, he bypassed the cell cycle entirely, pointing instead to the trehalose data. He revealed a long-dormant interest in the sugar, tracing back to his own postdoc days from 1973 to 1975 at ETH Zürich. In a beautiful closing of the scientific lineage, it turned out John had been warmly welcomed to Switzerland during that time by none other than Andres Wiemken. It was also during this Swiss interlude that John forged a lifelong friendship with Paul Nurse, a perfect reminder of the deep, happy coincidences of our field. In a moment that felt like being drafted into the major leagues, Pringle asked on the spot if I would join his lab as a postdoc. It was a once-in-a-lifetime opportunity to train in one of the world’s most prestigious environments. However, the final “peer review” was domestic; I returned to Michèle, who had joined me in Vienna. She didn’t hesitate, envisioning her own sabbatical in English literature at UNC Chapel Hill, an experience that would later blossom into her much-loved career as a high school English teacher. With her enthusiastic “yes”, the deal was sealed. We were going to America.

## The Tar Heel transition: oil leaks and insider jokes

### An ungraceful dark stain

We arrived in Chapel Hill in February 1994, greeted not by the warmth of the American South, but by a nasty flu and a persistent gray chill. Though I would later learn that March in North Carolina brings “spring fever” and shorts weather, our introduction was shivering and miserable. The transition was further complicated by our first major purchase: a cheap car that immediately marked its territory, and our arrival, by leaking its entire oil reservoir into John Pringle’s garden. As I was told later, that dark stain on his lawn outlasted my postdoc, a persistent, geological reminder of the somewhat ungraceful start to our American adventure.

### Verify your tools before questioning your talent

In those pre-internet days, the Atlantic Ocean felt like a wall of silence. However, a beacon of encouragement appeared early on via a fax from Stefan Hohmann, a collaborator with whom I would work for years on the trehalose-6-phosphate synthase/phosphatase complex. His message, welcoming me to this new world, bridged the gap between my past and future, giving me a warm sense that I hadn’t been entirely cut off from the scientific roots I left behind. I needed that warmth, because my first three months in the lab were a lesson in humility. I had arrived with the intention of cloning septin genes, a fascinating family of cytoskeletal proteins that John had essentially pioneered the study of, for a two-hybrid analysis, the cutting-edge method of the time. What followed was a quarter-year of absolute failure. I performed hundreds of mini-preps, burning through more than three liters of STET solution (at 0.5 ml per prep, the math was depressing). My self-confidence plummeted, though my determination held. The salvation came abruptly: we discovered the facility had synthesized my oligonucleotides incorrectly. With new primers, the experiments finally ran with the precision expected. Those three months were not lost; they taught me a core lesson: always verify the integrity of your tools before questioning the integrity of your talent.

### The “Hot-Lab” culture and the vocabulary wars

Once the work began to flow, life in Chapel Hill became, simply put, the best time of our lives. The atmosphere in the Pringle lab was distinct, vibrant, and incredibly supportive. We shared space with the lab of Tom Petes (specifically, we were in the isotope or “Hot lab”), which added another layer of genetic thoroughness to our regular discussions. My daily education was in the hands of my bench mate, Douglas (“Doug”) J. DeMarini (Fig. 7C and D), a fantastic colleague who graduated from the University of Wisconsin-Madison and took it upon himself to instruct me in the nuances of American culture and language. Determined to live up to his standards, I began devouring Time and Newsweek magazines. One afternoon, after yet another repetitive DNA prep, I turned to him and declared the work “stultifying”, a word I had just harvested from my reading. Doug stared at me blankly; he didn’t know the word. I was triumphant. I had out-vocabularized the native speaker. I ran to the rest of the lab, shouting, “I teached Doug a new word!” My glory went down the drain in an instant of grammatical carnage, but the laughter that followed defined the era.

### You’ve got the wrong yeast

**Figure 8:**
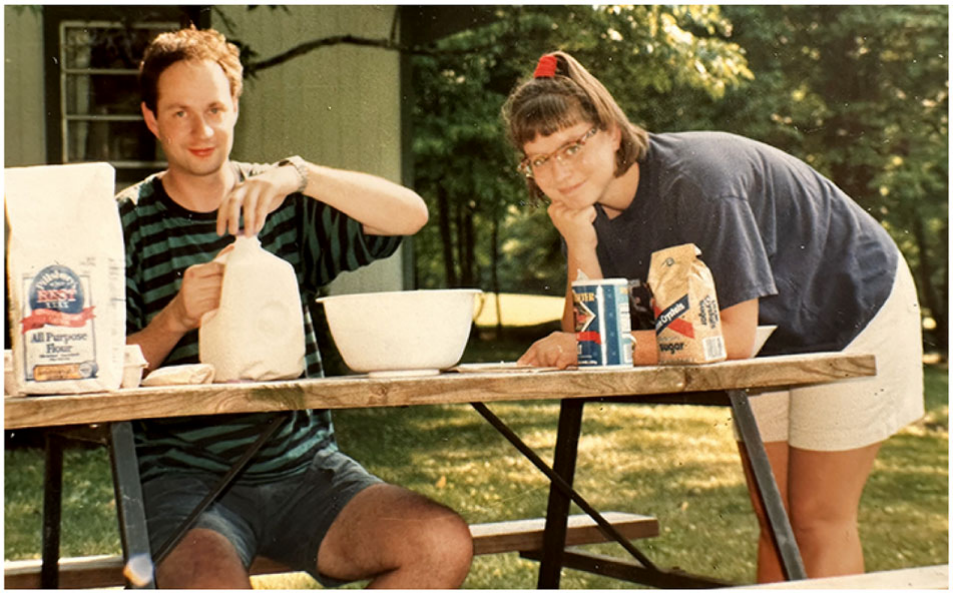
Jürg Bähler and Michèle baking bread with *S. cerevisiae—*a rare and delicious moment where Jürg finally abandoned his “wrong” yeast for the right one.

We worked hard, but we played just as hard, beach volleyball, indoor hockey, and especially soccer, inspired by the UNC women’s team, which was busy winning national championships. The lab felt even more like home when Jürg Bähler, a fellow Swiss from Hünibach, joined us (Fig. 8). Our friendship, forged over pipettes and insider jokes, has lasted to this day. Jürg is now the director of the UCL Institute of Healthy Ageing in London and leads a group at the Francis Crick Institute. In the lab, he would often tease me for my unwavering devotion to *S. cerevisiae*, so I naturally have to tease him right back about his one fatal flaw: he (not me) works with the “wrong” yeast (*Schizosaccharomyces pombe*).

### The Olympic standard of mentorship

Presiding over this organized chaos was John Pringle (who would later move his renowned lab to Stanford University). He was a model of leadership I have tried to emulate ever since: articulate, precise in his arguments, and a master of the written word. Having spent time in Switzerland himself (at the ETH), he was culturally attuned to my background and always supportive. John was also a dedicated mentor in the art of communication. We had regular scientific meetings in the Research Triangle Park with groups from Duke (where Joe Heitman had recently set up his own lab) and Raleigh. Before these events, John would train me 1:1, refining my delivery until my talk was as sharp as his own (or close). But our bond went beyond the bench. We shared a passion for sports, often spending weekends in the lab debating the Tour de France or swimming history. John had been an elite swimmer himself. In fact, after winning two silver medals at the 1963 U.S. Nationals, he briefly considered training for the 1964 Olympic team. However, holding an offer to begin his Ph.D. at Harvard, he instead chose the laboratory over the Olympic pool.

### A major heist

This shared passion led to one of my most memorable non-scientific victories. John offered me and Michèle tickets to see the “Tar Heels” play basketball in the legendary Dean Dome. Our seats were high up in the nosebleeds (Fig. 7B), but we noticed an empty bench right next to the court. Figuring it was worth a shot, we moved down. Inevitably, security swept through, chasing everyone away from that prime spot—except us. A guard at the end of the bench took one look at us, or perhaps heard our Swiss accent, and signaled that we could stay. We watched the rest of the game from the courtside, feeling like we had pulled off a major heist.

### Bridging the gap—with family

**Figure 9:**
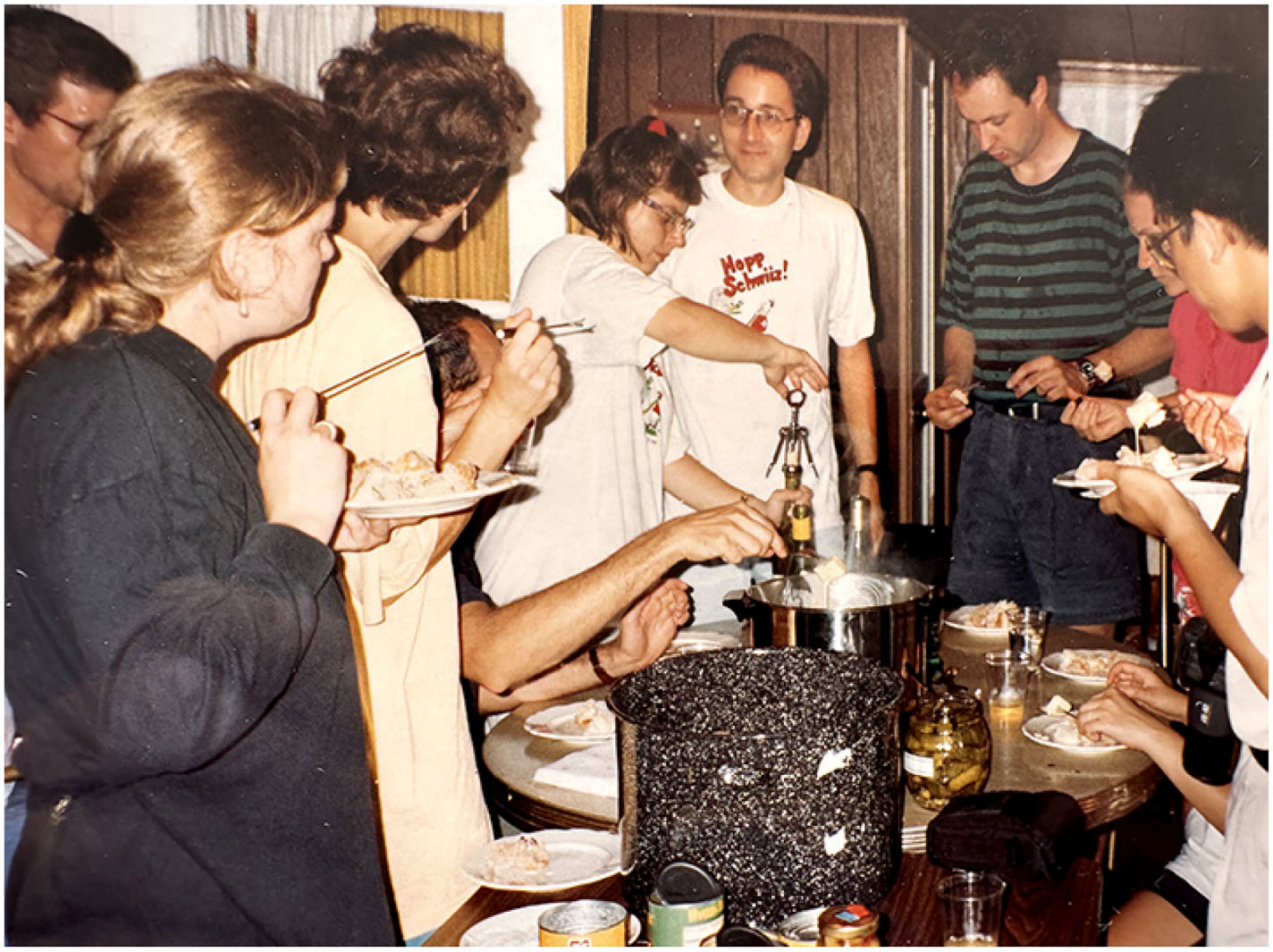
The “Swiss Connection” in the Appalachians: defying the 30°C heat and 100% humidity to prepare a traditional fondue, a true testament to our cultural resilience. Pictured surrounded by the rest of the lab (from left: Michèle opening the wine, me, and Jürg Bähler).

Scientifically, the persistence with the two-hybrid system finally paid off. After the initial struggles, I made steady progress identifying a new septin, but another meaningful contribution came when I provided a critical piece of the puzzle for the signalling pathways governing polarity. Using the two-hybrid system, I demonstrated that the Rho GTPase Cdc42 physically interacts with the PAK-family kinases in yeast. This data served as the “molecular glue” that substantiated the functional analyses being conducted by our collaborators. In August 1995, this synergistic effort culminated in two landmark publications where I was proud to share authorship. In a paper led by the lab of Steven Reed, my interaction data supported their discovery that Ste20 requires Cdc42 binding to drive the pheromone signalling pathway. Simultaneously, in a paper with the lab of Kim Nasmyth, we showed that the same interaction regulated Cla4 (a newly identified Ste20 homolog), driving the core process of cytokinesis. It was a powerful proof of the collaborative spirit John fostered. My bench work didn’t just serve our lab; it bridged the gap for two major biological stories, allowing us to publish alongside leaders in the field to reveal how a single GTPase could direct such diverse cellular fates. But the most lasting impression from Chapel Hill wasn’t the papers; it was the spirit. In the Pringle lab, I learned that a group of scientists could be a family (Fig. 9), and that science thrives best not in isolation, but in a community of shared curiosity.

## The Return: Hard landings, Basel, and the Geneva Crucible

### Lifeline in a hard landing

Everything comes to an end. Standing in front of Fordham Hall on a crisp day in 1995, I said goodbye to Doug. I remember shedding a tear as I walked away, knowing that the “Tar Heel” chapter was closed. The next chapter would be back in Switzerland, but the transition would prove to be a brutal reality check. I returned to a job market that seemed completely indifferent to my American success. I sent out more than 100 applications to pharmaceutical companies; I received zero offers. Academic positions were equally scarce. Just as the panic began to set in, my former mentors, Thomas Boller and Andres Wiemken, threw me a lifeline. They offered me the chance to return to the Institute of Botany to teach the plant physiology course, which I genuinely loved, and provided me with the lab space to start building my own research group. I will always be grateful for that safety net; it gave me the breathing room to transition from a postdoc to an independent thinker.

### A bitter rejection and a sweet kinase

However, independence brought its own trials. My very first grant application to the SNF was rejected. If I recall the comments correctly, the reviewers were sceptical of “yeast genetics”, a stinging critique given my recent training. But looking back, that rejected grant contained a promising seed that I simply couldn’t harvest at the time. I had proposed a suppressor screen for the temperature-sensitive *tps2* mutant. We knew the mutant died because of the toxic accumulation of trehalose-6-phosphate. My hypothesis was that if I could find a protein (regulator) that, when overexpressed, suppressed trehalose-6-phosphate production, I could cure the cell. I carried out the screen despite the rejection and isolated a candidate kinase. I figured out that it worked by inhibiting UDP-glucose pyrophosphorylase (Ugp1), thereby cutting off the supply of UDP-glucose, a precursor for trehalose-6-phosphate. I couldn’t carry the project to its full conclusion, but the logic was sound. Years later, Jared Rutter’s group would identify this same kinase as PAS kinase, confirming that it directly phosphorylates Ugp1 to control sugar flux. Seeing that mechanism published was a bittersweet validation: I had missed the paper, but my scientific instinct had been right on the money.

### We found the yeast cell’s brain

Undeterred, I wrote a second grant proposal. This time, I focused on Rim15, a kinase (and ortholog of the mammalian Greatwall kinase) I had earlier found to be defective not only in trehalose production but in the general entry into quiescence (G0) upon starvation. This marked the beginning of my long-standing fascination with how cells decide to stop dividing and survive. I was generously assisted by Niels Bürckert, a talented technician who had been with us since the early trehalose days. The heavy lifting on the molecular characterization was done by Anke Reinders, my very first PhD student, whose dedication was pivotal in setting the standard for the group. Together, we built the bedrock of the Rim15 story. We deduced that PKA must act upstream of Rim15, inhibiting it when glucose is abundant. This work culminated in our first major success, a publication in Genes & Development in 1998 (Reinders et al. 1998). But the story wasn’t finished. We found that treating cells with rapamycin induced the quiescence program, and this response was largely dependent on Rim15. The implication was massive: Rim15 was the integrator. It sat at the crossroads of the cell’s two most primary nutrient sensors, reading the glucose signal from PKA and the nitrogen/amino acid signal from TOR. We had found the brain of the cell’s survival strategy, proving that even a “hard landing” can lead to the most significant take offs.

### Over the bridge from the king of TOR

Based on this progress, I secured one of the prestigious SNF Professorship grants. Now, I needed a permanent home for this ambitious project. The logical choice seemed to be the Biozentrum, close to Michael Hall, the discoverer of TOR. In a stroke of luck, Peter Philippsen, the pioneer of *Ashbya* genetics, offered me lab space on the fifth floor in the newly built Pharmazentrum, which was connected directly to Hall’s floor in the Biozentrum by bridges. It seemed perfect.

### The gift of hard truth

I requested an audience with Michael to present my project on Rim15. The discussion was clarifying, though not in the way I had expected. Michael was direct, perhaps more so than I was prepared for. He pointed out the inevitable perception problem: if I worked on the TOR pathway while sitting just down the hall from its discoverer, I would struggle to establish my own scientific identity. Furthermore, my major competitor would be just right across the bridge. At the time, I was naive, focused only on the excitement of the science. But in retrospect, his candor was a gift. He forced me to confront a hard truth: to truly follow the logic of my own data, I needed complete independence. I needed an environment where I wasn’t the “neighbor”, but a distinct voice in a new territory. Peter Philippsen and Howard Riezman tried to persuade me to stay and pursue my SNF-funded studies there. The decision was difficult, the consequences were personal and painful, but I realized I had to leave Basel.

### The pain of a weekend husband and father

**Figure 10:**
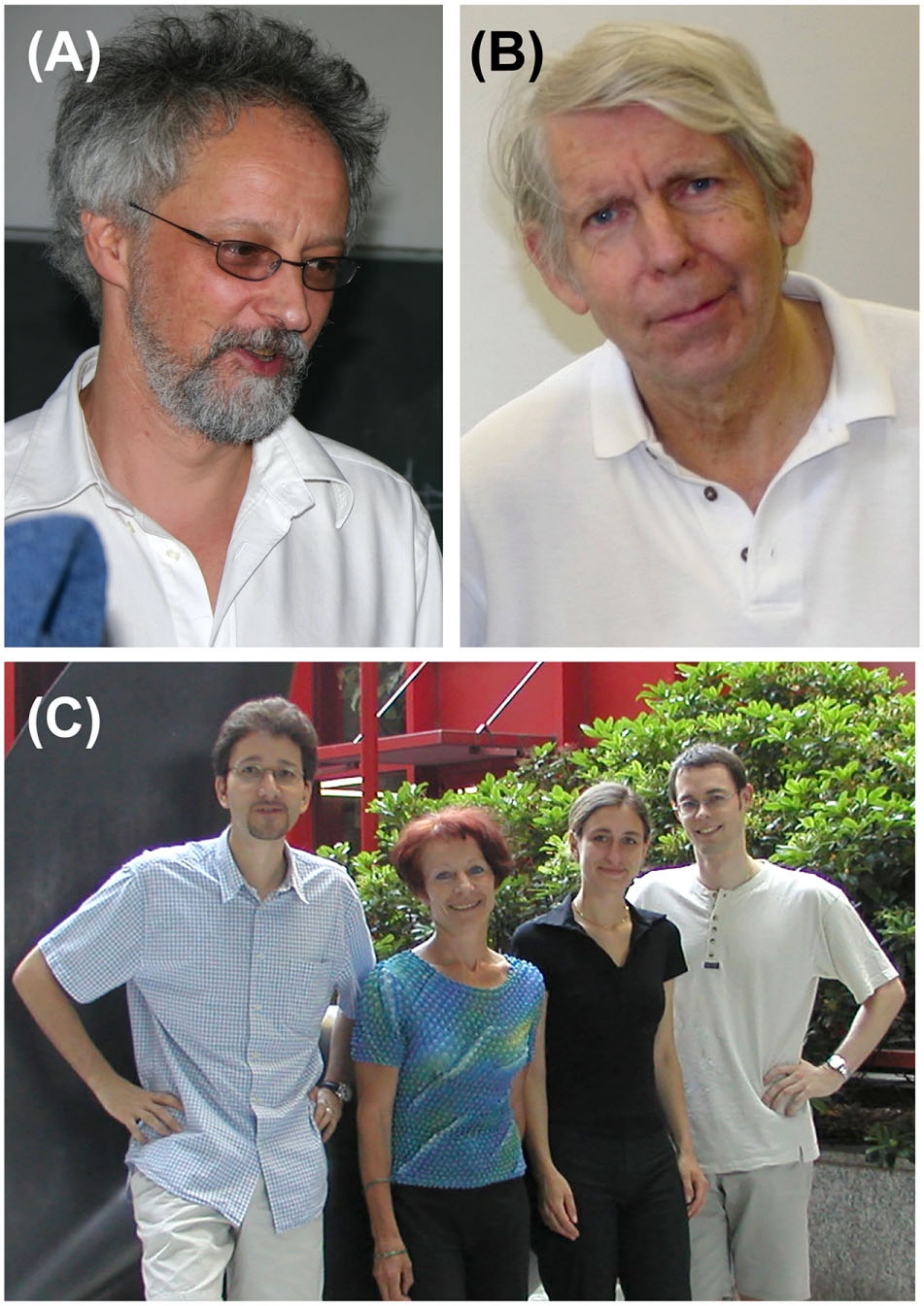
Patrick Linder (A), my former mentor who actively arranged the institutional groundwork for my transfer to the CMU in Geneva, alongside the ever-polite department head and distinguished British biochemist, Robin Offord (B). (C) Our early days in Geneva. Pictured in front of the CMU (from left: me, our lab technician Ruth Bisig, Frédérique Dubouloz, and Ivo Pedruzzi).

**Figure 11:**
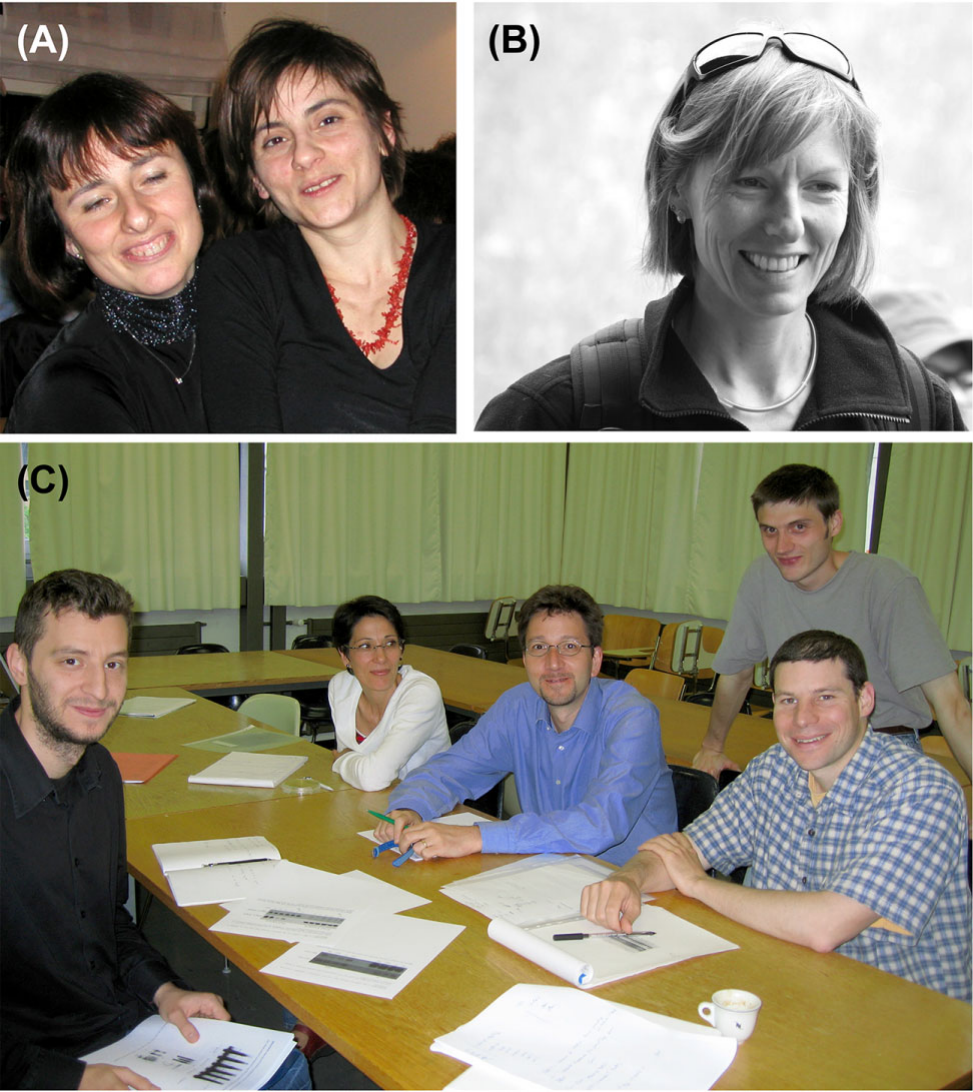
(A) Elisabetta Cameroni (right) and Valeria Wanke (left) sharing a joyful moment. (B) Malika Jaquenoud out on the trail. (C) Translating blots into breakthroughs: a collaborative session with my team in Fribourg (Matteo Binda on the left; opposite, from left: Marie-Pierre Péli-Gulli, me, and Robbie Loewith, with Gregory Bonfils behind Robbie), proving that science thrives best in a community of shared curiosity.

Salvation came from my former mentor, Patrick Linder (Fig. 10A), who had in the meantime moved to the Centre Médical Universitaire (CMU) in Geneva. When he learned of my situation, he didn’t just offer sympathy; he actively arranged the institutional groundwork to make my transfer possible, effectively paving the road to Geneva. However, uprooting my family was not an option. Michèle had her own career, and our daughter Fiona was only five years old. So, we made the hard choice that I would go ahead, alone. I was not entirely solitary in this professional migration. I was incredibly fortunate to work with Ivo Pedruzzi and Elisabetta Cameroni, who had joined me in Basel. In a brave decision for which I remain deeply thankful, they uprooted their own lives to follow me to the CMU in Geneva, ensuring that the science survived the move. Together with other brilliant additions to the team, such as Valeria Wanke and Frédérique Dubouloz, we forged a tight-knit group (Fig. 10C). The shared moments of joy and camaraderie were instrumental in balancing out the relentless pressures we faced during those early days, a spirit perfectly captured in a snapshot of Elisabetta and Valeria sharing a laugh (Fig. 11A). The scientific fit was perfect, but the personal cost was high. For a little over a year, I lived a divided life. I became a weekend father, working in Geneva during the week and commuting across the country to see my wife and little girl on weekends, leaving them behind every Sunday night or Monday morning. It was a lonely existence, fuelled only by the hope that the scientific gamble would pay off.

### Yeast in suits: my trojan horse for the biotech world

Parallel to my lonely struggle to establish a lab in Geneva, I found myself pulled into a rather surreal detour into the world of biotechnology start-ups. The spark was an institutional milestone: I had secured a patent for a heat-inducible promoter in *Hansenula polymorpha*, successfully licensed to Rhein Biotech. As one of the first patents to generate revenue for the University of Basel’s technology transfer office, it earned me the 2001 NETS (New Entrepreneurs in Technology and Science) Prize. The underlying premise of the prize was that the winners were aspiring entrepreneurs ready to launch their own start-up companies. There was just one problem: I had no product and no concrete business idea. To survive the program, I leaned on a collaboration I had started with Ernst Hungerbühler at the *Fachhochschule beider Basel* (FHBB). We were using baker’s yeast and its endogenous reductive dehydrogenases to perform highly stereoselective asymmetric reductions of ketones, offering an ecologically friendly way to produce enantiomerically pure chiral compounds. I used this applied research to construct a fantasy start-up project. It was my trojan horse, an elaborate role-play that allowed me to peek behind the curtain of the biotech industry.

### The academic tourist: pitching profit margins with hand models

While the recognition stemmed from Basel, the award itself, an intensive entrepreneurship crash course at Babson College near Boston, pulled me away just as I was trying to get Geneva off the ground. Led by renowned business professors, the program served as a fascinating, bizarre distraction. Surrounded by fiercely ambitious peers hungry for the corporate hustle, I harbored my secret: I was an academic tourist. The program involved dynamic training sessions where we had to deliver business plans and mock elevator pitches to the American business instructors. I enthusiastically leaned into the role-play. I vividly remember standing at the front of the room, waving my hands around as makeshift molecular models, desperately trying to explain the concept of enantio-specificity to a training panel that primarily cared about my theoretical profit margins. My true identity was hilariously confirmed by a mandatory corporate personality assessment designed to evaluate our entrepreneurial aptitude. When my results came back, my “conscious persona” was officially classified as an “Observer/Reformer”. In the colourful world of corporate psychology, this meant I was analytical, deeply cautious, and driven by precise data rather than risk. The test had seen right through my pitch: I was unequivocally an academic.

### 235 million reasons I prefer the lab

That feeling of being a complete outsider in a high-stakes corporate world peaked when a successful entrepreneur took our cohort to an exclusive bar strictly open to millionaires. Taking in the unnecessarily fancy atmosphere, the absurdity was undeniable—none of us belonged there. Or so I thought. One peer in our group was developing a cancer immunotherapy spin-off called GlycArt. Just 4 years later, he would sell his company to Roche for 235 million Swiss Francs, permanently earning his seat at that bar. I watched the start-up world mint millionaires, but that “Observer/Reformer” profile was right. While my peers dreamt of securing actual venture capital and massive exits, I found myself longing for the precision of the laboratory.

### The Geneva years: catastrophic losses and worries

While my professional life was occupied with overcoming these abstract academic and corporate hurdles, my personal reality was abruptly overshadowed by profound grief. My time at the CMU was a crucible, both personally and scientifically. The period was bracketed by deep loss. Just as I started in Geneva, my father died. A couple of years later, my younger brother passed away unexpectedly. And shortly after I left Geneva for my next position in Fribourg, I would lose my mother. It was a time of overwhelming emotional weight, a loneliness compounded by the weekly separation from my family. Professionally, the stakes were equally high; for the first 3 years, I was unable to publish a single paper from my own lab. The clock was ticking, and even the department head, the ever-polite Robin Offord (Fig. 10B), a distinguished British biochemist who had come to Geneva from Oxford and had previously collaborated with the legendary Frederick Sanger, two-time Nobel Laureate, took me aside to gently express his “worry” that I had yet to deliver.

### Accepted: freedom to look where others were not and see things they didn’t see

**Figure 12:**
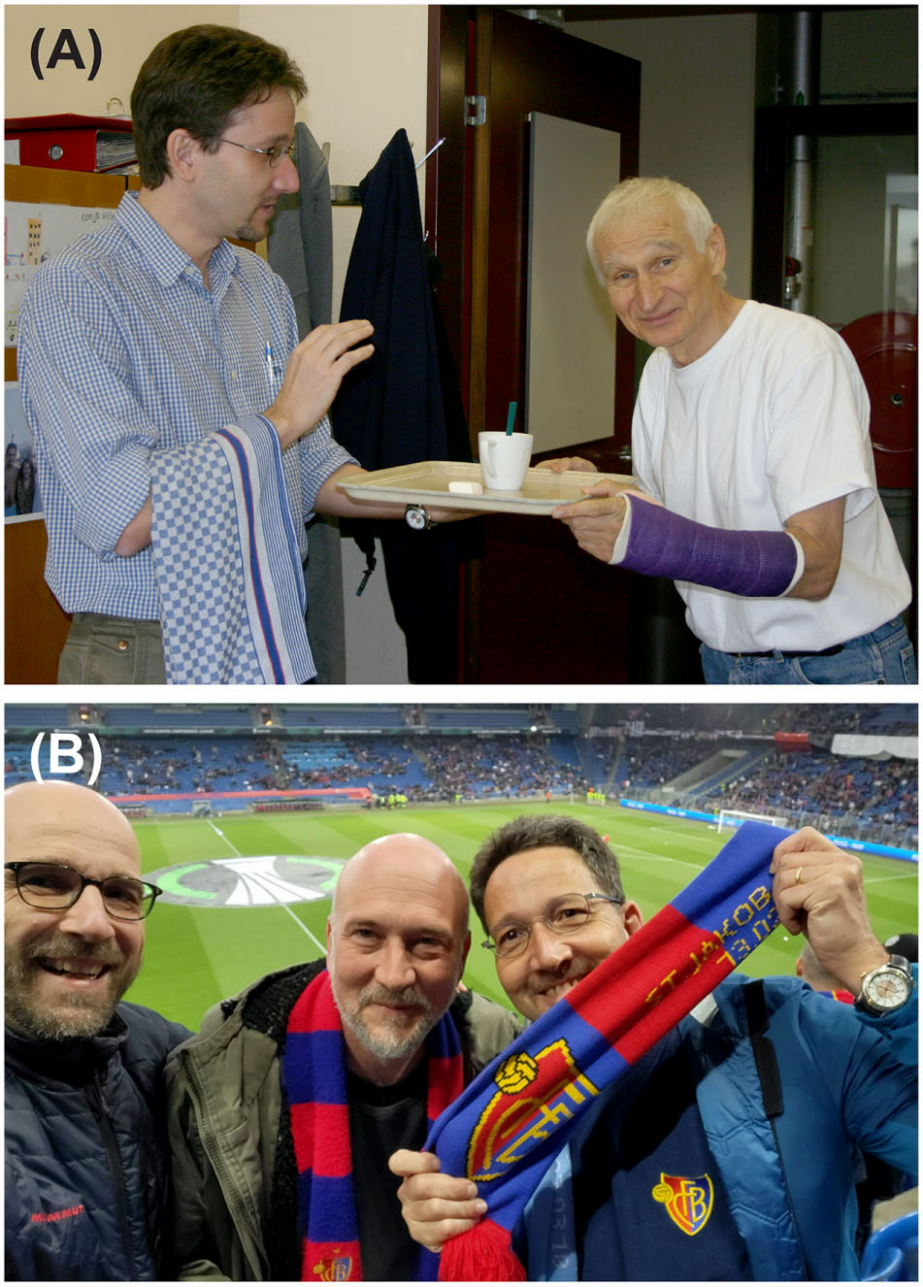
(A) Serving a well-earned coffee to Costa Georgopoulos—mentorship from a “grandmaster of chaperone biology” doesn’t come for free. (B) FC Basel vs. Fiorentina semi-final at the “Joggeli” Stadium in Basel, 18 May 2023, with Jörn Dengjel (left), Dieter Kressler (center), and me. Everything is a matter of positioning, timing, and teamwork.

**Figure 13:**
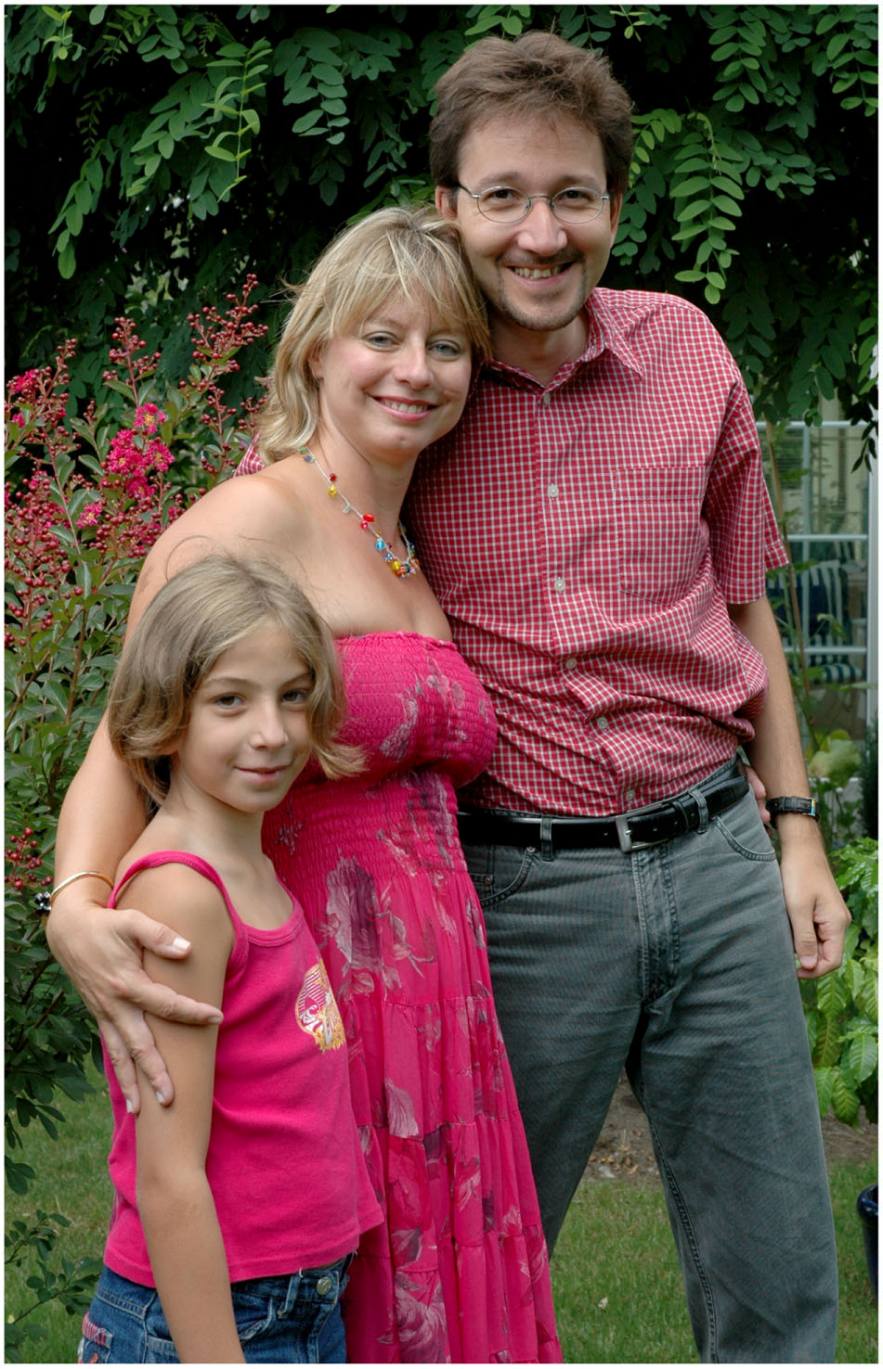
My family reunited in Geneva; my daughter, Fiona, my wife Michele, and me.

What kept me afloat during this “dark night of the soul” was the incredible support system at the CMU. Patrick Linder remained a constant ally. I also found a new mentor in Costa Georgopoulos (Fig. 12A), a true grandmaster of chaperone biology (famous for pioneering the discovery of the GroE chaperone machine) who trained in the lab of Nobel Laureate Salvador Luria. Costa supported me with resources and a sense of humour that proved vital when the grief, or the data, seemed insurmountable. What also kept me afloat during this difficult period was a pivotal change in my personal life. In 2002, after a little over a year of living a divided life, my family finally joined me in Geneva. This long-awaited reunion provided the indispensable emotional grounding I needed to push through the scientific hurdles (Fig. 13). The turning point finally arrived in the fall of 2003, in the form of a single email. We had submitted our manuscript detailing how Rim15 integrates PKA and TOR signalling, the culmination of years of work and the ultimate validation of my move to Geneva. I remember staring at the screen, afraid to click, knowing that the “Accepted” or “Rejected” status of this one paper would likely determine if I could continue in academia. When I finally opened the message to see the word “Accepted”, it was more than a publication; it was the realization that our scientific gamble had paid off (Pedruzzi et al. 2003). I immediately ran to find the paper’s first author, Ivo Pedruzzi, to share the moment. In hindsight, the distance and the struggle were the catalysts for our success. Had I stayed in Basel, I might have been constrained by prevailing current of thought; forced into the isolation of Geneva, I had found the freedom to look where others were not.

### Waking the sleeper: the EGO complex

While the Rim15 story secured my academic survival, a parallel line of inquiry would define the next phase of my research. I became fascinated by the flip side of the coin: if Rim15 helps the cell enter quiescence, what controls the exit? How does the cell “wake up” when conditions improve? Logic dictated that TOR must play a central role in this resurrection. To prove it, we undertook a powerful genetic screen using the entire yeast knockout collection. The idea was simple: we treated the cells with rapamycin to induce a deep growth arrest, then washed the drug away and watched to see which mutants failed to recover. We coined these the *ego* mutants (standing for exit from growth arrest).

### The electric jolt, ignition key, and command center

Analysing these mutants led us to the vacuolar membrane-localized EGO complex, containing the Rag GTPases (Dubouloz et al. 2005). This result introduced a completely new concept: a dedicated, membrane-bound scaffold that controlled TOR at the vacuole to sense nutrients. We had found the missing link. If the TOR kinase was the engine of cell growth, the EGO/Rag GTPase complex was the ignition key. There it was again, that electric jolt of discovery I had longed for since the Tps2 days in the cold room. We hadn’t just found a gene; we had found a location and a mechanism. We had discovered that the vacuole was not just a storage bin, but the command center for growth control. This finding established a universal blueprint for nutrient sensing, providing the basis for what would later be recognized as a fundamental principle of eukaryotic biology.

### The stabilizing force and the catalyst

In 2005, when Robbie Loewith joined the Department of Molecular and Cellular Biology in Geneva, what began as a professional acquaintance quickly evolved into an enduring friendship and an open, natural collaboration that benefited both our labs (Fig. 11C). We were immediately on the same wavelength, and our bond soon extended beyond the laboratory as we became close personal friends, often meeting with our families. This relationship provided a stabilizing force for me; Robbie was the person who could help redirect my “crazier” ideas while sharing the daily burdens of managing a competitive field and the complexities of the editorial system. We established common lab retreats to exchange ideas freely, without the guardedness often found in high-stakes research.

### The assay we all needed

Robbie’s arrival was also a catalyst for the entire field; by identifying Sch9 as the first *bona fide* TOR target in yeast, he provided the tool we all needed to finally measure TOR activity. This was a breakthrough that allowed the whole field, including my own group, to finally quantify the effects of the nutrient-sensing machinery we were uncovering. To this day, we continue to brainstorm and organize the joint retreats that began during those Geneva years, maintaining the natural spirit of discovery that first brought our groups together.

## The move to Fribourg: a new intellectual home

### This organism has four legs

By 2007, it became clear that the CMU in Geneva, despite the unwavering personal support of mentors like Patrick and Costa, could not be my permanent home. It was a medical faculty, and the institutional bias against non-mammalian model organisms was often palpable. I recall being summoned by a renowned local professor to witness a mouse operation, a gesture that felt less like a clinical invitation and more like a gentle reminder of the local hierarchy. As I stood there, he turned to me and said in French, “Tu vois Claudio, ça c’est un organisme qui a quatre pattes” (“You see Claudio, this is an organism that has four legs”). The subtext was clear: in the world of high-stakes medical research, yeast was occasionally viewed as a second-class citizen. I knew I had to move to an environment where my model organism was respected for its genetic power rather than judged by its anatomy.

### Curiosity the only currency that matters

I applied for a position at the University of Fribourg and was fortunate to be chosen. My arrival was smoothed by the enthusiastic support of Andreas Conzelmann, an expert in yeast lipid metabolism. Our joint lab meetings became a source of incisive feedback and scientific joy, proving that excellence is often found in the quality of the dialogue rather than the size of the university. He also bestowed upon me a parting gift of sorts: he organized for Malika Jaquenoud (Fig. 11B), a most talented technician, to integrate into my lab, ensuring we had excellent technical hands from day one. The transition was further eased by the loyalty of my team. Elisabetta Cameroni, who had already followed me from Basel to Geneva, moved once again to Fribourg. Her proactive spirit was instrumental; she often carried out experiments before I could even finish articulating the hypothesis. I was also joined by a talented senior researcher, Marie-Pierre Péli-Gulli, whose dedication is such that, to this day, she undertakes a 90-min commute to contribute to our science. In Fribourg, I finally found the intellectual home I had been seeking, a place where curiosity was the only currency that mattered.

### The universal blueprint: EGO at 20

**Figure 14:**
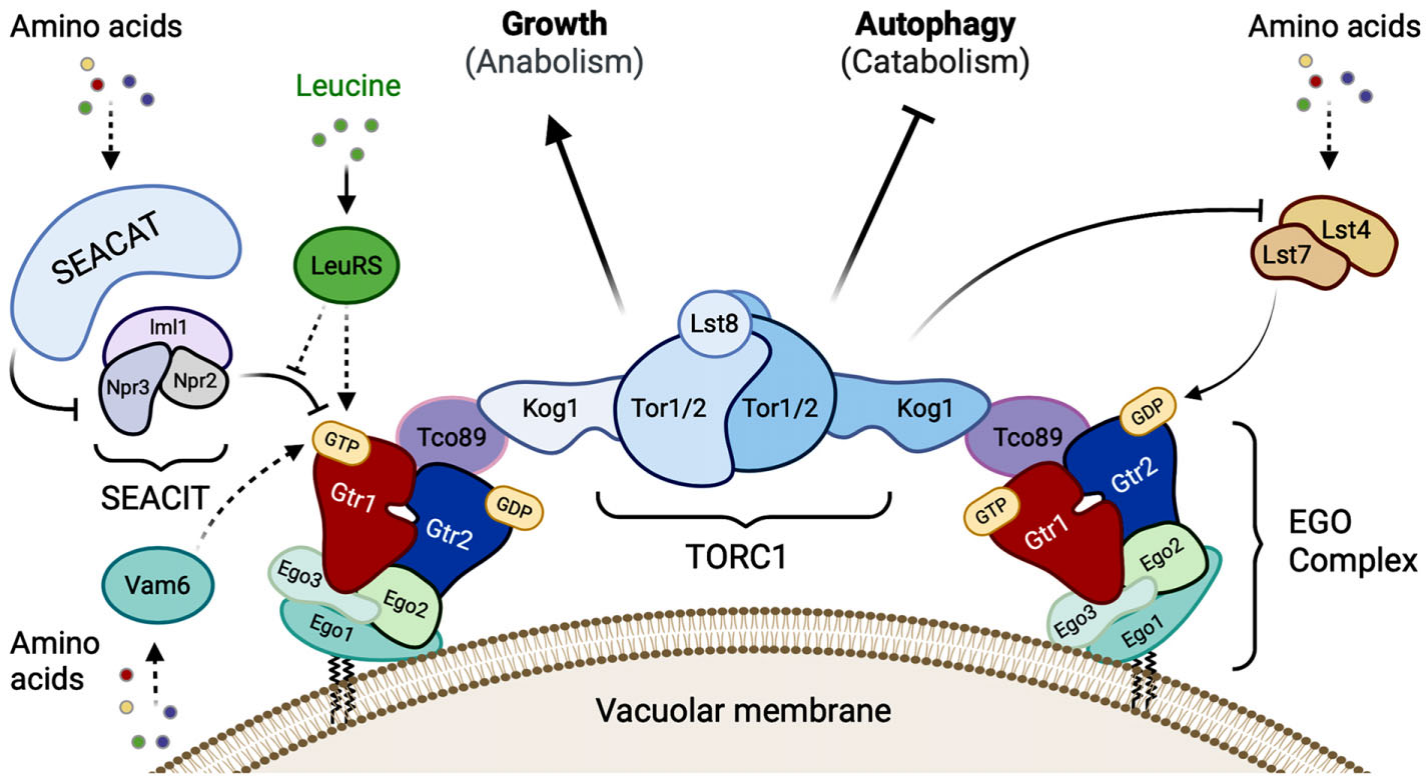
Twenty years of EGO: deciphering the cellular nutrient-sensing blueprint. This schematic encapsulates two decades of our laboratory’s work mapping the architecture of vacuolar TORC1 regulation. By systematically identifying the central EGO/Rag GTPase command center alongside its surrounding regulatory network, including the SEACIT and Lst4/Lst7 GAP complexes, the upstream SEACAT regulator, the LeuRS nutrient sensor, the Vam6 activator, and the Tco89 tether, we established the foundational framework that now defines our understanding of growth control across all eukaryotes (for details, see De Virgilio 2026b). Created in BioRender. De Virgilio, C. (2026) https://BioRender.com/5tn33qk.

With this team in place, our science flourished. In terms of scientific output, we were quite successful. Over the years, we mapped the amino acid sensing pathway in yeast, showing how the EGO complex activates TORC1 (Fig. 14). But the most gratifying realization was that this machinery wasn’t unique to yeast. It is highly conserved in higher eukaryotes. The mechanism we uncovered in simple baker’s yeast turned out to be the same blueprint used in human cells, a subtle confirmation that our work had touched on a fundamental principle of life. This journey is detailed in my recent review, “EGO at 20: the Rag GTPase-TORC1 nutrient sensing blueprint” (De Virgilio 2026b).

## The Fribourg spirit: science, soccer, and friendship

### From the lab to the pitch—positioning, timing, and teamwork

Fribourg offered the perfect environment for this work. It is a small, bilingual (French-German) university town that lacks the intimidating aura of the ETHZ or the Biozentrum, but it possesses a quiet excellence. The absence of cutthroat competition allowed us to do research in a more relaxed, yet highly productive way. This atmosphere attracted exceptional colleagues. In 2014, Jörn Dengjel joined us. Beyond establishing a world-class proteomics platform and pioneering work on autophagy, Jörn became an essential confidant and a vital scientific partner. Then there is Dieter Kressler, a fellow “disciple” of Patrick Linder and Ed Hurt. Known for his beautiful work decoding ribosome biogenesis and his discovery of the dedicated chaperones that safely escort aggregation-prone ribosomal proteins, Dieter is far more than a colleague; he is my daily grounding presence. As natives of Basel, we are bonded by our dry humor and our loyalty to FC Basel. From our daily lunches to our traditional “Burger Fridays”, both have become dear friends and indispensable parts of my life in Fribourg. Together, we have formed a research hub where the pursuit of knowledge is a daily source of joy. Our collaboration extends seamlessly from the bench to the pitch (Fig. 12B). I have always loved soccer, from the fields of St. Johann to the intramural games in Chapel Hill. In Fribourg, we play Futsal. I play in attack, still hunting for goals, while Jörn anchors the defense. Our car rides home are often spent dissecting the details of the games we won or lost, debating strategy with the same intensity we apply to our signalling data. There is a beautiful symmetry in this; whether in the lab, at the stadium, or on the court, success is a matter of positioning, timing, and teamwork.

### Is someone pushing on the door?

While our daily life in Fribourg was defined by a supportive and collaborative spirit, the external validation of our research often came through high stakes encounters on the global stage. One such defining moment occurred back in 2012, when I received an invitation that felt like a summons to the Conclave of our field: a meeting at Les Treilles. This exclusive gathering in the south of France is where the architects of the TOR field convened every 4 years or so. Invited at the last minute to replace a colleague, I arrived feeling the distinct pressure to prove I belonged. I chose to present our most significant unpublished work: the discovery of the SEACIT/SEACAT complex, the upstream regulators of the EGO complex. The presentation sparked immediate interest, leading to a discussion with David Sabatini about coordinating our findings in Science. It is unclear to me whether or not he had the mammalian counterpart of SEACIT/SEACAT in hand at that point. However, what followed was a crash course in the “geopolitics” of high-stakes publishing. While our manuscript faced a challenging review process, the competing mammalian story moved forward. It felt, for a moment, as if the door to our own discovery was being closed from the other side.

### TOR de France—redemption from the physicians

Thankfully, the editorial team at Science Signaling emerged to restore the balance. Recognizing the fundamental value of our yeast data, they ensured our findings were published just days before the competing mammalian story (Panchaud et al. 2013). For years, the fact that the “GATOR” nomenclature became the standard citation felt like a semantic concession, but time has a way of clarifying one’s priorities. In October 2025, during the “TOR de France” meeting in Nice, I experienced the defining professional redemption. I sat in the audience and watched as clinicians described treating “GATORopathies” with rapamycin. The nomenclature had changed, but the map we had drawn in yeast remained the guide. Seeing our basic genetic work in yeast translated into life-saving clinical interventions for real patients provided a satisfaction that no citation count could ever match. While we laid the groundwork in the “lower” eukaryotes, I credit the mammalian field for the essential work of translation. It is a beautiful demonstration of how curiosity-driven research, even when born from the heat of competition, ultimately converges to serve human life.

### The heartbeat of the lab: curiosity, freedom, and Friday afternoons

Reflecting on the output of the laboratory over the decades, I realize that our most significant discoveries often emerged not from rigid adherence to a grant proposal, but from the unscripted curiosity of my students. My philosophy has always been to provide a “safety net” of funding and support, and then to step back and allow the students the freedom to follow the data. I encouraged them to pursue “Friday afternoon experiments”, high-risk ideas that often yielded the most exciting insights. This approach required a specific type of laboratory culture, one defined by mutual support rather than internal competition. I have always believed that a happy lab is a productive lab, fueled by the sense of fun and the drive to ask ‘why’ for the sake of knowing that together define the heartbeat of this profession. By simply focusing on shared curiosity and mutual support rather than internal competition, I’ve had the privilege of mentoring a remarkable group of young scientists. It has been a quiet joy to see so many of my doctoral students—many of whom naturally happened to be brilliant women—go on to forge highly successful careers of their own. Seeing them thrive, whether running their own labs or leading teams in the biotech sector, remains as satisfying as any paper we ever published.

### Beyond the bench: a journey of resilience

**Figure 15:**
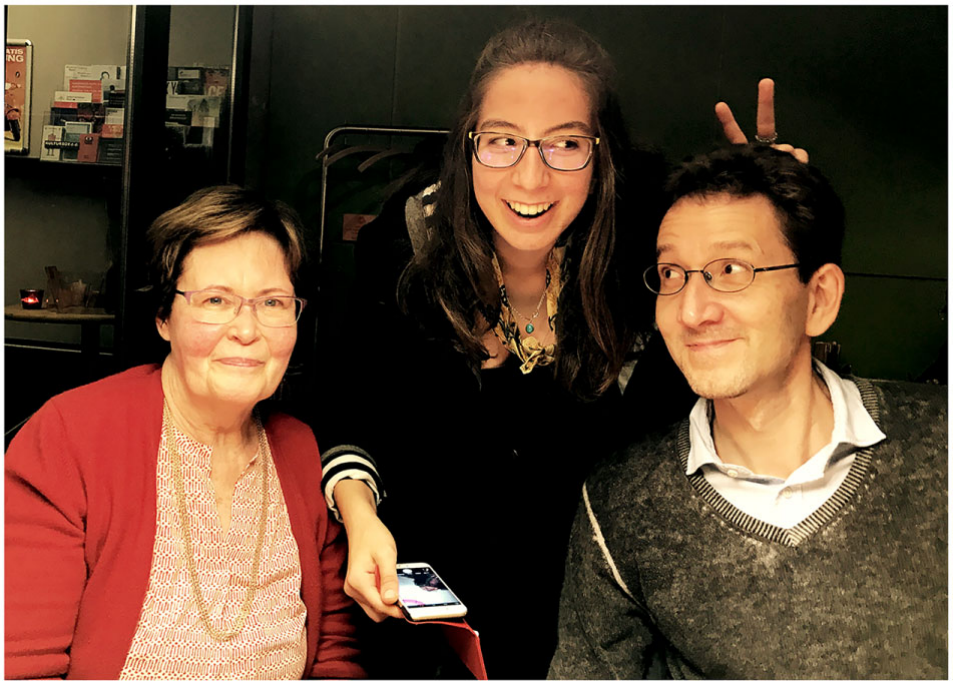
Family dynamics at play during Fiona’s 20th birthday. Fiona playfully flashes “bunny ears” behind my head, ensuring I don’t take myself too seriously. We are joined by my stepmother, Ursula, who provided the stability of my own teenage years.

Life outside the laboratory, of course, continued its unpredictable march. You may wonder what became of the “little girl” who, together with Michèle, saw me off every Sunday evening during those long commuting times to Geneva. I am happy to report that she grew into a remarkable woman. Although I taught her to master “*Desoxyribonukleinsäure*” as one of her very first words, she eventually charted her own course. She has always possessed a vibrant spirit and a wonderful sense of humour that keeps me from taking myself too seriously, a dynamic wonderfully caught in a family photo where she playfully flashes “bunny ears” behind my head, joined by my stepmother, Ursula, who provided the stability of my own teenage years (Fig. 15). Fiona found her calling away from the bench, successfully passing the rigorous *numerus clausus* to study speech therapy after her Bachelor’s degree. Today, she brings her empathy and specialized training to her work in childcare. Her own path has not been linear, but she has faced her challenges with a resilience that deeply humbles me. Watching her step into her life’s work with such quiet confidence, strength, and grace is, for me, the most rewarding discovery of all.

### Epilogue: the circle of curiosity

**Figure 16:**
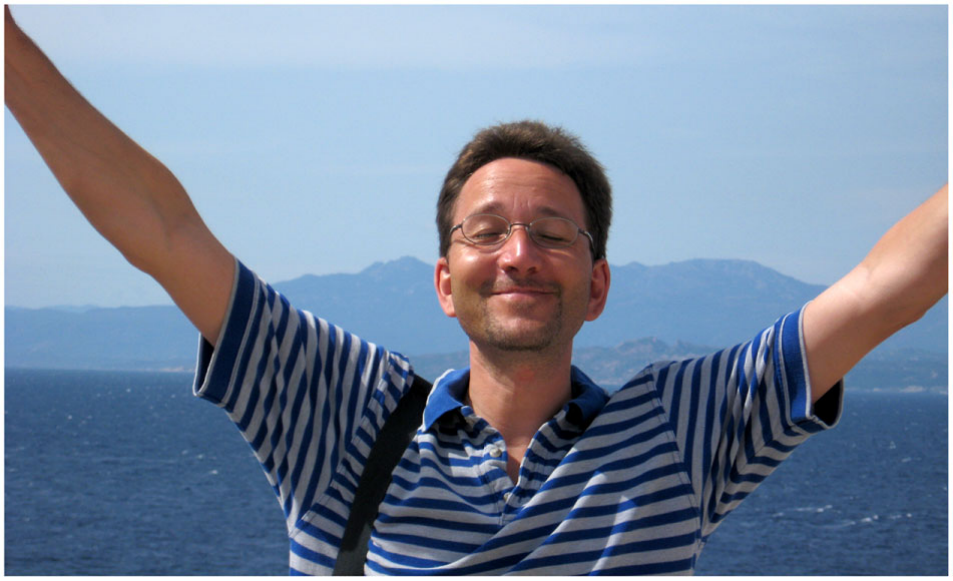
No longer in the shadow of the gate—life is good.

As I look back, I realize my journey has traced a remarkable historical circle. I am a citizen of Bologna, home to the first university in the Western world (1088). I am a proud citizen of Basel, home to the first university in Switzerland (1460). And I completed my formative training at UNC Chapel Hill, the first public university in the United States. From the industrial streets of St. Johann to the quiet valleys of Fribourg, my path has been driven by a single, simple engine: curiosity. I started my life playing in the shadow of a medieval gate, oblivious to the science hidden around me. Decades later, I find myself studying the molecular gates, the TOR complex and the EGO signaling hub, that determine the fate of a cell. The scale has shifted from the architectural to the microscopic, but the thrill of discovering what lies behind the gate remains exactly the same. Today, stepping out from the shadow of that childhood landmark and standing in the sun, I can confidently say: life is good (Fig. 16).

**Figure 17:**
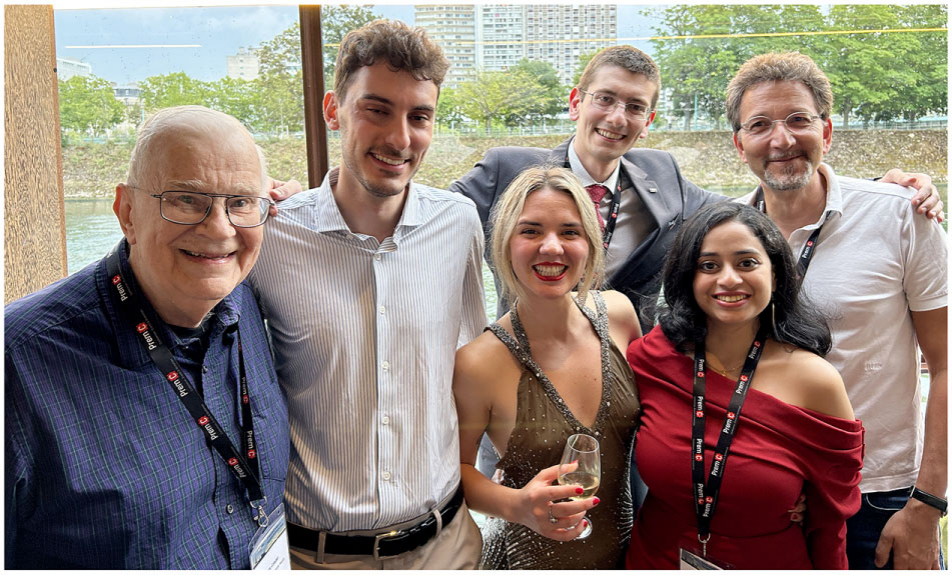
Terry and my crew on a mission, navigating the high seas of ICYGMB32 in Paris (from left: Terry, Marco Caligaris, Rebecca Calviello, Cyril Jaggi, Saloni Koli, and me).

### You are my constant—thank you

I am deeply grateful to the mentors who protected me, the colleagues who challenged me, and most of all, the students and postdocs who trusted me with their careers. In the end, science is often described as a cold search for objective truth, but I have found it to be a deeply human search for connection: connecting molecules in a pathway, connecting data points to a theory, and connecting with the brilliant, diverse human beings who walk this path with you. And finally, to Michèle, who sat by the Rhine with me when I should have been in a practical, and who has walked every step of this winding path by my side: thank you. The models and events may change, but you remain my anchor, my constant, and the love of my life.
